# Toward Decarbonization of the Titanium Industry via Hydrogen Plasma Smelting Reduction

**DOI:** 10.1002/advs.202514689

**Published:** 2025-10-20

**Authors:** Laura Gabriela Torres‐Mejia, Ubaid Manzoor, Guangyi Guo, Chris W. Bumby, Dierk Raabe, Isnaldi R. Souza Filho

**Affiliations:** ^1^ Max Planck Institute for Sustainable Materials 40237 Düsseldorf Germany; ^2^ Robinson Research Institute Victoria University of Wellington Lower Hutt 5010 New Zealand; ^3^ The MacDiarmid Institute for Advanced Materials and Nanotechnology Victoria University of Wellington Wellington 6012 New Zealand; ^4^ Institut Jean Lamour CNRS (UMR 7198) Université de Lorraine Nancy F‐54000 France

**Keywords:** carbon‐free metallurgy, hydrogen plasma reduction, ilmenite processing, iron production, sustainable materials, Titanium ores, titania‐rich compounds

## Abstract

Surging global demand for titanium, driven by its essential role in aerospace, transportation, chemical industries, and as a white pigment, is intensifying pressure on scarce high‐grade ores, like rutile, leading to their rapid depletion. As a result, ilmenite (FeTiO_3_) mineral sands are emerging as the cornerstone of future titanium supply. However, conventional ilmenite processing routes are energy‐intensive, carbon‐emissive, and require multiple reduction and refining stages. Here, a single‐step hydrogen plasma reduction process is introduced that simultaneously produces two critical materials for a sustainable economy, high‐purity iron and a Titania‐rich compound, directly from low‐grade ilmenite concentrates, without fossil fuels and direct CO_2_ emissions. This process unifies smelting, reduction, and refining (of both iron and Titania oxide mixture) in one operation, at rapid kinetics and selective impurity removal. Silica, an impurity usually removed in a separate chemical step, is reduced by ≈75% within the proposed plasma‐based operation, enabling direct downstream use of the Titania‐rich compound. The resulting iron achieves purity high enough for direct use in steel production, while the Titania‐rich compound serves as an upgraded CO_2_‐free precursor for titanium metal or pigment production. This work introduces a zero‐carbon metallurgical route for maximizing value from low‐grade ilmenite while advancing decarbonized industrial metals processing.

## Graphical abstract

Hydrogen plasma smelting reduction enables direct, zero‐carbon conversion of low‐grade ilmenite into steel‐grade iron and a titania‐rich precursor for pigment production. The single‐step process merges smelting and refining, sharply lowers silica content, and offers a breakthrough path for sustainable, high‐value extraction from abundant mineral sands.

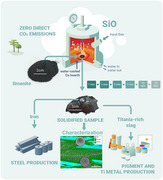

## 1 Introduction

Titanium is an essential strategic metal due to its exceptional strength‐to‐weight ratio, corrosion resistance, and ability to outperform most other metals under extreme conditions. These properties make it indispensable for applications in aerospace, medical devices, automotive, chemical manufacturing, and energy conversion. The global titanium market was valued at USD 24.7 billion in 2021 and is projected to reach USD 33.5 billion by 2026.^[^ ^1^ ^]^ In the automotive sector, the demand for titanium metal has been projected to reach 123 000 metric tons by 2027^[^ ^2^ ^]^ as lightweight materials become more critical for electric vehicles. Overall, the global titanium consumption is projected to reach **284,000 metric tons** by 2027,^[^ ^2^ ^]^ reflecting its expanding and essential role in advanced technologies and infrastructure. In addition, a related product, titanium dioxide (TiO_2_) is a widely used white pigment in the paints and coatings sector. Pigment accounts for ≈95% of global titanium‐bearing minerals production, and ≈78% of the total value of the broader titanium market. The market for titanium dioxide pigments is anticipated to increase from USD 10.3 billion in 2021 to USD 14.1 billion by 2026.^[^ ^1^ ^]^

**Figure 1:**
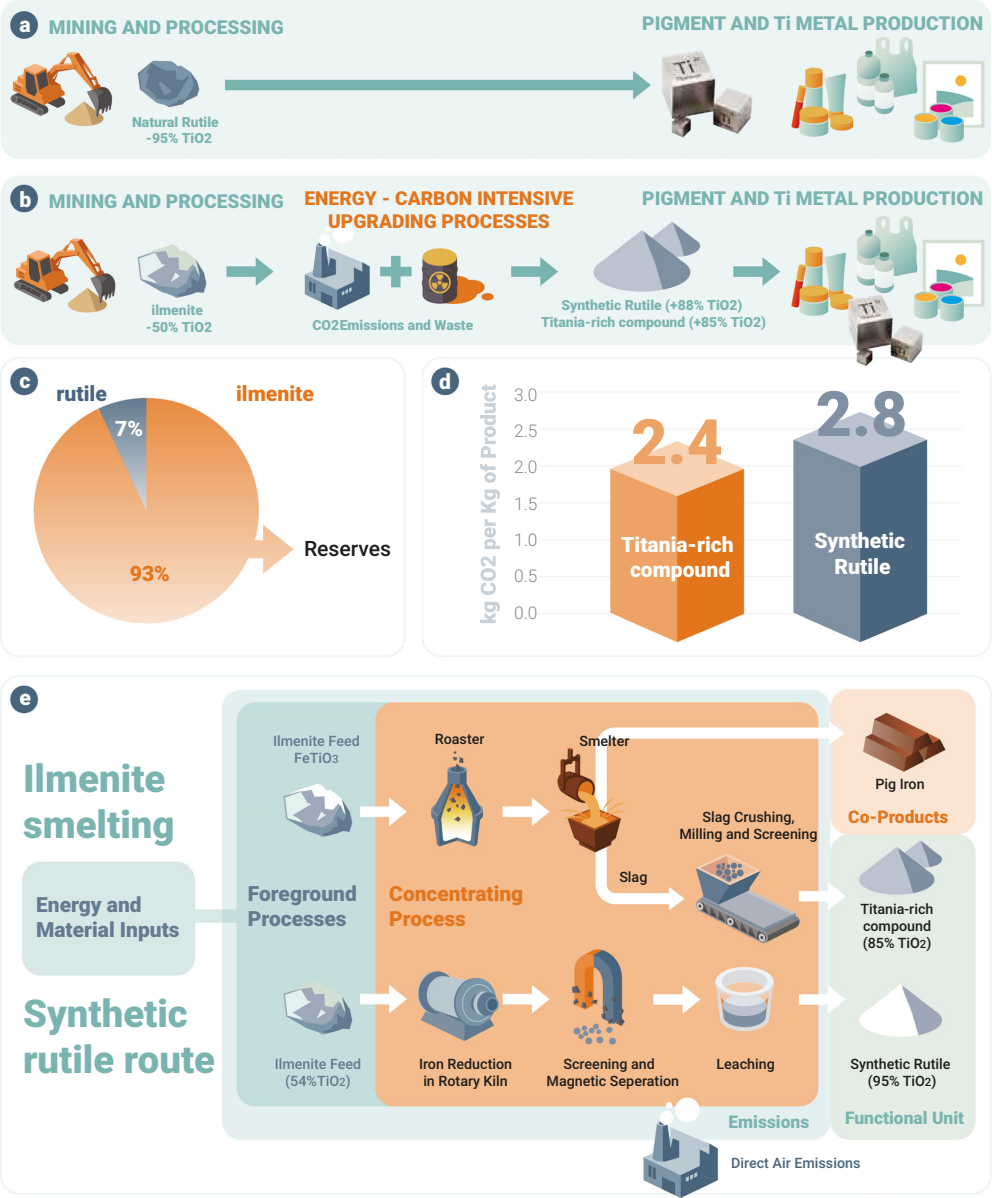
Overview of the titanium refining industry. a) Mining and processing of natural rutile. b) Mining and processing of ilmenite. c) Current reserves of ilmenite and natural rutile.^[^ ^10^ ^]^ d) CO_2_ emissions associated with the production of Titania‐rich compound and synthetic rutile.^[^ ^15^ ^]^ e) Current processing routes for ilmenite include smelting and the synthetic rutile pathway, both of which rely on carbon‐based reducing agents, resulting in significant CO_2_ emissions.^[^ ^15^ ^]^

Whilst essential for these key applications, titanium refining remains a notoriously energy‐intense, expensive, and CO_2_‐intense process. Within naturally‐occurring minerals, the element titanium is typically found in conjunction with oxygen and iron, mainly in the form of two key minerals: rutile (TiO_2_) and ilmenite (FeTiO_3_).^[^ ^3^ ^]^ Historically, the high purity mineral rutile (≈95% TiO_2_), has been directly utilized as feedstock for the production of TiO_2_ pigment and raw titanium metal^[^ ^4^ ^]^ (**Figure** **1**a). This can be refined to <100 ppm of contaminant metals using the chloride or sulphate processes^[^ ^5^ ^]^ before being reduced to a metal product using the Kroll^[^ ^6^ ^]^ or Hunter^[^ ^7^ ^]^ processes. However, natural rutile deposits are scarce^[^ ^8^ ^]^ and have been significantly depleted in recent decades, due to surging market demand (Figure 1c). As a result, the global titanium industry has shifted to utilizing ilmenite as its primary feedstock.^[^ ^9^ ^]^ Today, ilmenite satisfies more than 95% of the worldwide demand for titanium‐bearing minerals, with global production reaching 8.6 million metric tons of ilmenite in 2023.^[^ ^10^ ^]^

However, ilmenite mineral ores typically contain only 30–60 wt.% TiO_2_, due to their high iron content, along with other elements. This cannot be fed to a pigment or metal refining process directly, and instead must first be upgraded to an intermediate product containing ≈85%TiO_2._^[^ ^11^ ^]^ There are two types of globally traded Ti‐bearing intermediate materials, known as ‘synthetic rutile’(SR) and ‘upgraded titania slag’(UGS), respectively, depending on the upgrading process used (see Figure 1b).

*Synthetic rutile* is produced in a 2‐step process. First, a carbothermic direct process reduces ilmenite to iron metal and residual oxides, usually in a rotary kiln or fluidized bed at 850–900 °C using coal or other fossil carbon sources as reductant.

(1) $$2C_{\left(s\right)}+O_{2\left(g\right)}\to 2CO_{\left(g\right)}\;\left(\mathrm{partial}\;\mathrm{combustion}\right)$$

(2) $$\mathrm{FeTi}O_{3\left(s\right)}+\mathrm{CO}\to Fe_{\left(s\right)}+\mathrm{Ti}O_{2\left(s\right)}+CO_{2\left(g\right)}\;\left(\mathrm{direct}\;\mathrm{reduction}\right)$$

The iron is then hydrometallurgically leached from the solid product as a waste, leaving behind a Ti‐rich oxide, which typically contains >90 wt.% TiO_2_.^[^ ^12^ ^]^ This is referred to as ‘synthetic rutile’.

Alternatively, a Titania‐rich compound colloquially known as *Upgraded titania slag (UGS)* can be formed via a smelting process in which ilmenite concentrate and coal are heated together in an electric arc furnace at temperatures above 1600°C.^[^ ^13^, ^14^ ^]^ Carbothermic reduction occurs such that liquid iron metal is formed along with a Titania‐rich compound:

(3) $$\mathrm{FeTi}O_{3\left(l\right)}+C_{\left(s\right)}\to Fe_{\left(l\right)}+\mathrm{Ti}O_{x\left(l\right)}+CO_{2(g)}\;\left(\mathrm{Carbothermic}\;\mathrm{smelting}\right)$$

The liquid metal and titania‐rich compound mutually separate as immiscible layers in the melt, and are then separately tapped to produce both iron and upgraded titania‐rich compound, the latter with a typical TiO_2_ content of ≈85%(see Figure 1e).

The elevated levels of TiO_2_ within SR and the Titania‐rich compound (compared to the initial ilmenite) mean that these intermediates can then both be used as valuable feedstocks for the same processes used to refine the natural mineral rutile.^[^ ^16^ ^]^ However, conventional processes for producing either SR or Titania‐rich compound are highly CO_2_ intensive, due to the extensive use of fossil carbon as reductant. In both cases, more than 2 tonnes CO_2‐e_ are emitted per tonne of product (see Figure 1d).^[^ ^15^ ^]^ Also, the use of fossil carbon as a reductant in conventional ilmenite smelting introduces several impurities into the iron phase, including silicon (0.5–2 wt.%), carbon (3–5 wt.%), sulfur (0.02–1 wt.%), and phosphorus (0.1–1 wt.%). These impurities must be removed through additional high‐temperature refining steps before the iron can be used in steelmaking, significantly increasing the energy demand and complexity of the conventional process. Simultaneously, the titania‐rich compound produced retains most of the silica present in the original ilmenite ore. This high silica content is detrimental in downstream processing, as it increases acid consumption, complicates filtration, and reduces titanium recovery efficiency, thereby making the process both more energy‐ and chemically intensive.

Decarbonizing the titanium supply chain will therefore require the development of alternative reduction technologies that eliminate fossil carbon as a reducing agent and effectively manage impurity levels to enable cleaner, more sustainable upgrading of ilmenite ores. Similar challenges related to high CO_2_ emissions and impurity‐laden iron are also faced by the steel industry, where hydrogen‐based reduction has been proposed as a promising route to significantly reduce carbon emissions.^[^ ^17^, ^18^, ^19^ ^]^

Numerous studies have explored the hydrogen direct reduction (DR) of ilmenite in the solid‐state, focusing on phase transformations, kinetics, and impurity effects.^[^ ^20^, ^21^, ^22^, ^23^, ^24^, ^25^ ^]^Si et al.^[^ ^20^ ^]^ found that reducing Panzhihua ilmenite at 1150°C with 100 mL/min H_2_ for 80 min achieved 87.5% metallization, producing Fe, M_3_O_5_ (M = Mg, Ti, Fe), and titanium oxide. Lu et al.^[^ ^21^ ^]^ showed that MgO impurities slow the reduction, with natural ilmenite forming metallic Fe and rutile at 900 °C, and ferrous pseudobrookite above 1000 °C. Lv et al.^[^ ^22^ ^]^ improved reduction rates by pre‐oxidizing ilmenite before H_2_‐Ar reduction at 850–950 °C. Dang et al.^[^ ^24^ ^]^ conducted thermogravimetric studies on the isothermal and non‐isothermal reduction of Panzhihua ilmenite. Under isothermal conditions (873–924°C), the main products were iron, TiO_2_, and MgTiO_3_. In contrast, non‐isothermal conditions at 1220°C allowed for further reduction of TiO_2_ and MgTiO_3_ into metallic iron and Mg_x_Ti_3‐x_O_5_ (0.45 < x < 1). Chen et al.^[^ ^25^ ^]^ highlighted that higher H_2_ content and temperature enhance reduction kinetics.

However, the direct reduction of ilmenite pellets using hydrogen gas has several key challenges.^[^ ^23^, ^25^, ^26^ ^]^ One of the primary difficulties is the impact of impurities such as MgO, MnO, and SiO_2_, which are commonly found in natural ilmenite. These impurities hinder the reduction of Fe^2+^ into metallic Fe as they form Mn and Si oxide‐enriched zones, preventing the complete reduction of Fe^2^⁺, leading to lower metallization degrees.^[^ ^23^ ^]^ Another challenge arises from the pellet structure and its interaction with the reducing gas. The dense structure of roasted pellets can impede gaseous diffusion, thereby reducing the overall reduction kinetics.​^[^ ^25^ ^]^ Additionally, prolonged reduction times of up to 40 min are required to achieve only a 65% degree of metallization.^[^ ^26^ ^]^

In this context, a highly promising alternative is hydrogen plasma smelting reduction (HPSR). Plasma‐smelting addresses several of the aforementioned bottleneck challenges by utilizing highly energetic and reactive hydrogen species (H, H⁺). This approach directly addresses the primary goal of ilmenite smelting, namely, to increase the TiO_2_ content by reducing Fe oxides in a carbon‐free, single‐step, pyrometallurgical process.^[^ ^17^, ^19^ ^]^ The application of hydrogen plasma enables efficient reduction, as the reactive hydrogen species significantly accelerate the reduction of Fe‐oxides, resulting in enhanced metallization degree and kinetics.^[^ ^27^, ^28^, ^29^ ^]^

**Figure 2:**
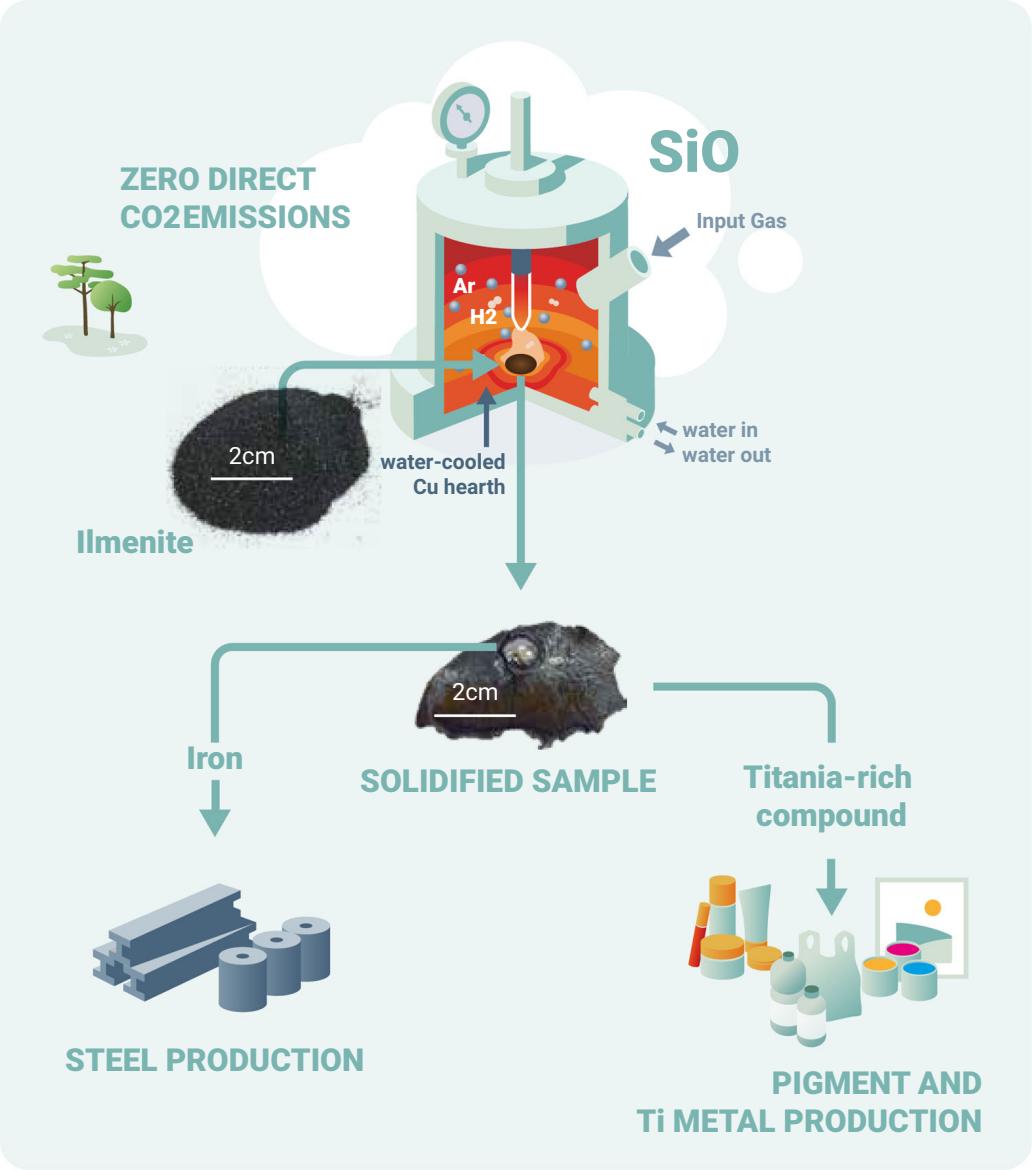
Hydrogen plasma smelting reduction process (HPSR). Hydrogen plasma smelting reduction process (HPSR), the ilmenite concentrate is placed in the water‐cooled copper hearth, and the chamber is flooded with an Ar‐H_2_ mixture, this process yields, titania‐rich compound as valuable feedstock for the titanium industry, highly pure iron, and gaseous water as by product with zero direct CO_2_ emissions.

Here, we report laboratory experiments on the HPSR of two different ilmenite ore concentrates. This approach yielded two high‐purity products: metallic iron suitable for direct integration into steelmaking processes, and a titania‐enriched compound suitable for further refining to titanium products (**Figure** **2**). HPSR of ilmenite eliminates the need for additional pre‐treatment steps and avoids the direct generation of CO_2_ emissions, aligning with the global push toward decarbonizing metallurgical industrial processes.

## 2 Results and Discussion

### 2.1 Original Ilmenite Concentrates

Two different variants of beneficiated ilmenite mineral sands were sourced from mine sites in the South Island of New Zealand. These are referred to as LGI‐1 and LGI‐2 – where “LGI” stands for ‘low grade ilmenite’. Microstructural and chemical characterization of these raw ilmenite concentrates was performed using X‐ray diffraction (XRD), scanning electron microscopy (SEM), energy dispersive X‐ray spectroscopy (EDS), and X‐ray fluorescence (XRF). The elemental composition of each sample is shown in **Table** **1**.

**Table 1:** Composition of the as‐received ilmenite concentrates. As‐received ilmenite concentrates composition determined by XRF. In this measurement, the iron content is quantified as Fe^3+;^ however, it should be noted that this value includes iron present in both the +3 and +2 oxidation states.

	Fe_2_O_3_	MnO	TiO_2_	CaO	K_2_O	SO_3_	P_2_O_5_	SiO_2_	Al_2_O_3_	MgO	Na_2_O
LGI‐1	45.7	1.7	38	1.1	0.2	0.15	0.2	8.2	3.88	0.46	0.4
LGI‐2	38.8	2.1	29	3	0.25	<0.01	0.2	19	6.78	0.66	0.2

**Figure 3:**
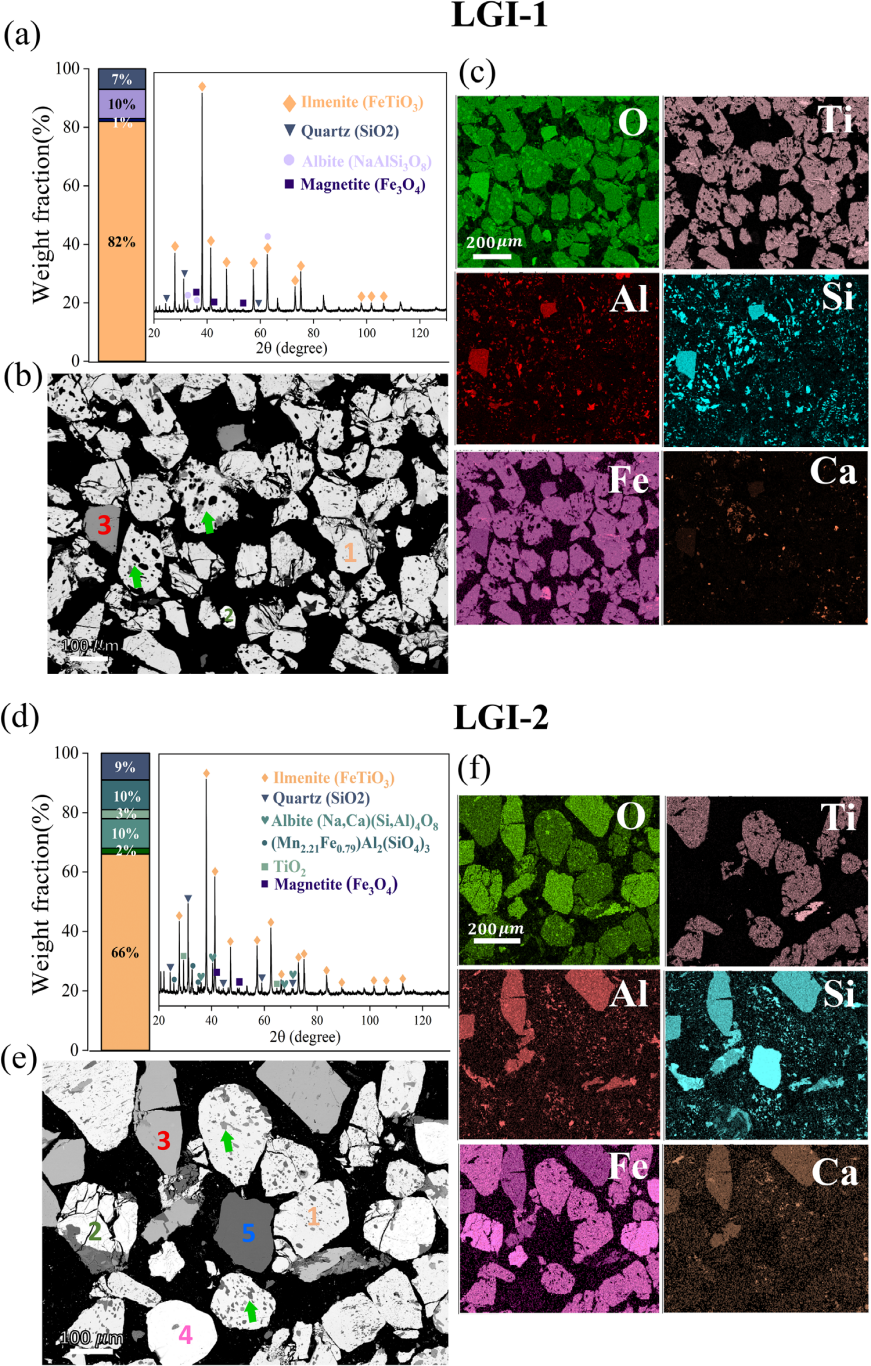
Initial characterisation of the as‐received ilmenite mineral sand concentrates. a, d) Weight fraction of the main phases identified by XRD in the LGI‐1 and LGI‐2, and XRD patterns with the corresponding labelled peaks. b, e) Microstructure of the particles contained in the LGI‐1 and LGI‐2 concentrates; number 1 corresponds to ilmenite (FeTiO_3_), 2 corresponds to magnetite (Fe_3_O_4_), 3 corresponds to garnets (silicate minerals, e.g., [Mg, Fe, Mn]_3_Al_2_(SiO_4_)_3_),4 corresponds to Zirconia (ZrSiO_4_) and 5 to Quartz (SiO_2_). The green arrows point out the intergrowth of the ilmenite grains with silicate‐based domains. c, f) Elemental distribution maps of the as received concentrates.

XRD analysis (**Figure** **3**a,d) to contain titanium and iron in the form of ilmenite (FeTiO_3_), accounting for 67% and 83% of the total weight, respectively. The remaining crystalline minerals are mainly SiO_2_, TiO_2_, and other silicates.

Figure 3b,e show the microstructure of the particles contained in both LGI‐1 and LGI‐2 concentrates, respectively. With the aid of the corresponding elemental distribution maps shown in Figure 3c,f, we can identify each type of particle contained within the ilmenite concentrates. In Figure 3b,e, the white particles are identified as ilmenite (marked as number 1 in orange). They are intergrown with dark domains corresponding to a silicate‐based phase (as pointed out by green arrows in Figure 3c,d). Magnetite (Fe_3_O_4_) is highlighted in green, as number 2. Brighter grey particles, labelled as number 3 (in red), represent garnets (silicate minerals, e.g., [Mg, Fe, Mn]_3_Al_2_(SiO_4_)_3_), while particles marked with magenta (number 4) correspond to zirconia (ZrSiO_4_). Quartz (SiO_2_) appears as solid dark grey grains, labelled as number 5 (in blue). Based on the semi‐quantitative analysis provided by EDS, the Fe: Ti ratios of 1.5 and 1.3 were estimated for LGI‐1 and LGI‐2 concentrates, respectively. Both ratios are higher than the ideal stoichiometric ratio for ilmenite (viz., 1.1). This deviation is attributed to the presence of magnetite and Fe bearing garnets in the raw samples, as evidenced in Figure 3a,d. The average particle size of the grains inside the ilmenite concentrate is ≈183 µm for the LGI‐1, and 111 µm for the LGI‐2.

### 2.2 Fe Extraction through Hydrogen Plasma Smelting Reduction

**Figure 4:**
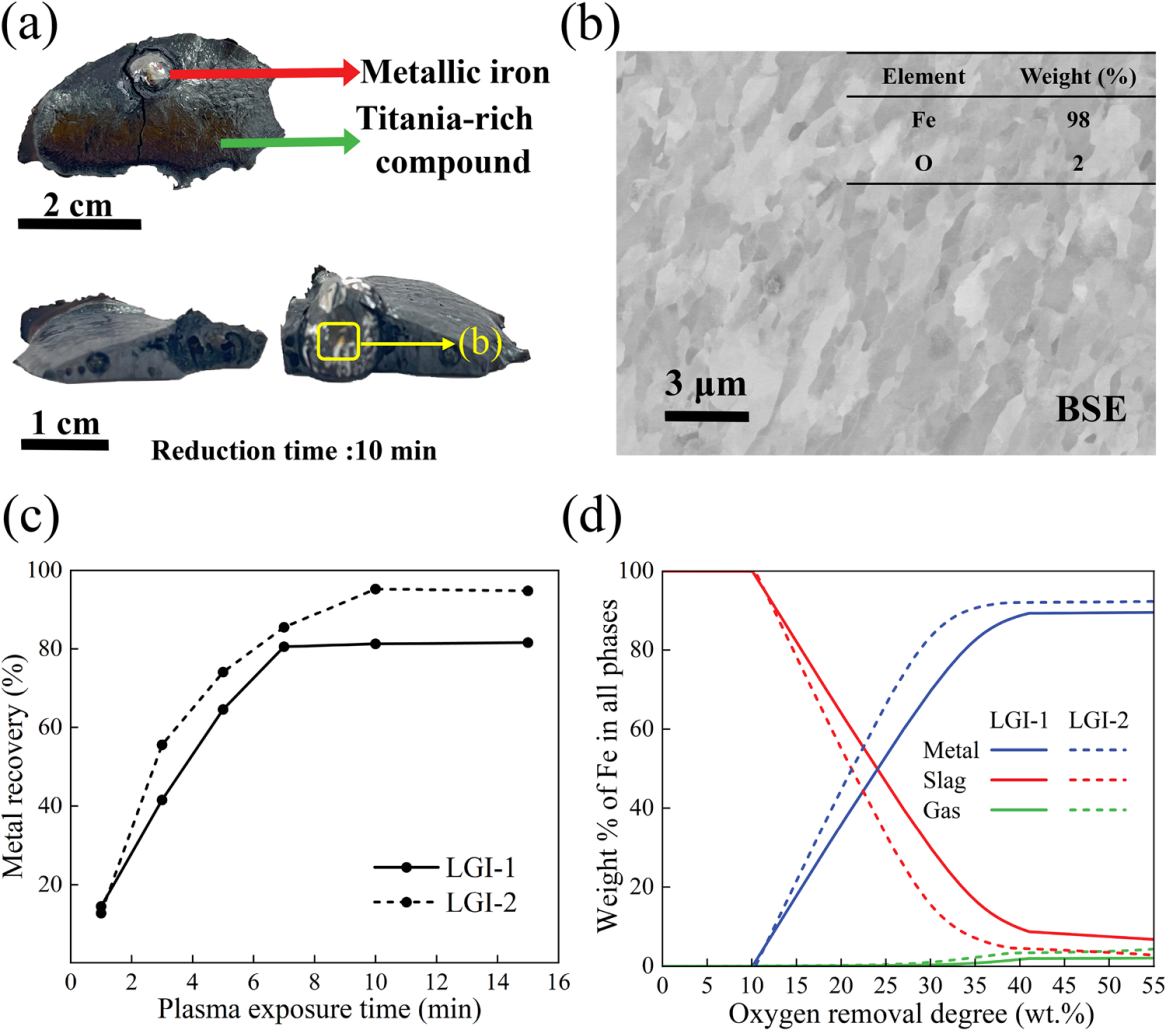
Metal recovery was obtained for both concentrates (LGI‐1 and LGI‐2). a) Top view and cross‐section of the solidified sample processed via HPSR for 10 min. The titania‐rich compound surrounds the metallic nodule, and the inner pores are due to gas entrapment. b) BSE‐SEM image of the metallic portion, a columnar structure is observed due to the fast cooling imposed by the water‐cooled copper hearth. c) Metal recovery obtained for both concentrates at different times of plasma exposure time, calculated as the ratio of metallic iron content in relation to the total iron content in the original input feedstock. The straight and dashed lines correspond to LGI‐1 and LGI‐2, respectively. A higher metal recovery is observed for LGI‐2. d) Thermodynamic calculations illustrating the weight % of Fe present in the metallic phase, the Titania‐rich precursor, and the gas as a function of the oxygen removal degree (straight lines correspond to LGI‐1, and dashed lines to LGI‐2).

Low‐grade ilmenite concentrates were processed under a lean hydrogen plasma arc (Ar‐10%H_2_) ignited at 200 A, as detailed in the Experimental section. The final product after reduction is composed by a metal portion (indicated by the red arrow in **Figure** **4**a) surrounded by a titania‐rich compound. The morphology of the metal portion evolves as the plasma exposure time increases. After 1 min reduction, an irregular nugget shape is observed, accompanied by smaller iron droplets distributed across the sample surface. As the exposure time increases, these particles coalesce to form a well‐defined, rounded shape metal particle that is located in the center of the sample. The iron droplets were cut, and the cross‐section was analyzed. The microstructure shows a columnar grain structure (Figure 4b), arising from the unidirectional solidification process, which is primarily influenced by rapid heat extraction from the water‐cooled Cu hearth on which the samples are processed. EDS analysis of the metallic iron specimen revealed a composition of 98% Fe (Figure 4b), with negligible impurities (<0.01%) of Ca, S, P, and Si. This high purity is particularly noteworthy, as it eliminates the need for additional refining processes. In contrast, iron produced via conventional carbothermal methods typically contains significant levels of P, S, and C, requiring further treatment. The resulting metallic nodules are therefore suitable for direct use in the steel industry, offering substantial energy savings by avoiding multi‐step refining procedures.

The reduction efficiency of the ilmenite concentrates was evaluated using the metallization value, calculated as the ratio of metallic iron content to the total iron content in the original feedstock (32% in weight % for LGI‐1, and 27% in LGI‐2). The sub‐micron sized metallic portions entrapped in the titania‐rich material were quantified via XRD, and the amount obtained was added to the one that had been magnetically extracted (see Experimental Section).

As depicted in Figure 4c, the LGI‐2 specimen exhibited a remarkable iron extraction efficiency, achieving a maximum extraction rate of 93% after 10 min of exposure to hydrogen‐containing plasma. This high level of extraction translates into the recovery of ≈3.3 grams of iron from the original feedstock, highlighting the effectiveness of the plasma reduction process for this concentrate. Conversely, the LGI‐1 specimen demonstrated a slightly lower maximum iron extraction of 81%, which was reached after 7 min of plasma exposure. This extraction rate corresponds to the recovery of 3.9 grams of iron from the initial feedstock.

Thermodynamic calculations for metal recovery from ilmenite concentrates were conducted for 1600°C and a hydrogen atmosphere using the software ThermoCalc. 15 g of molten ilmenite of each type of ilmenite concentrate, LGI‐1 and LGI‐2, were exposed to a controlled gas mixture of Ar‐10% H_2_ at a constant pressure of 900 mbar (see Experimental Section). Figure 4d shows the amount of Fe present in the metallic liquid (metal), oxide liquid (titania‐rich material), and the gas phase for both concentrates as a function of the oxygen removal from the molten material. The residual oxide mixture feedstock obtained from LGI‐1 exhibits a higher solubility for Fe (solid red line) compared to that of LGI‐2 (dashed red line). This higher solubility is translated into a diminished precipitation of Fe in the form of metal, as depicted by the blue lines in Figure 4d. These results agree with the results found experimentally, where LGI‐2 exhibits a higher metallization degree compared to LGI‐1, thus suggesting that the HPSR process proceeds near the thermodynamic equilibrium.

The thermodynamic stabilization of FeO–Ti_2_O_3_‐bearing slags is fundamentally constrained by the M_3_O_5_‐type stoichiometry, in which the oxide liquid assumes the generalized formulation: [(FeO, MgO, MnO).2(TiO_2_)]_x_.[(Ti_2_O_3_,Cr_2_O_3_,V_2_O_3_,Al_2_O_3_). (TiO_2_)]_y_, such a representation reflects the requirement that every divalent cation (Fe^2+^, Mg^2+^, Mn^2+^) must be compensated by two tetravalent Ti^4+^ ions, whereas each pair of trivalent cations (Ti^3+^, Cr^3+^, V^3+^, Al^3+^) must be electrostatically balanced by a single Ti^2+^.^[^ ^13^ ^]^

The **LGI‐2 concentrate** exhibits an enhanced reservoir of **non‐ferrous divalent species** (notably Mg^2+^ and Mn^2+^), which can isomorphically substitute for Fe^2+^ within the pseudobrookite solid solution. This preferential occupation of Fe^2+^ lattice sites by alternative divalent cations destabilizes Fe–O bonding interactions, thereby facilitating the **selective reduction and precipitation of metallic Fe** from the silicate matrix. In contrast, the **LGI‐1 system** is comparatively deficient in auxiliary divalent cations, constraining the extent of substitution. Consequently, Fe^2+^ remains structurally indispensable within the pseudobrookite to uphold the **M_3_O_5_ stoichiometric invariant**, thus suppressing iron liberation and stabilizing Fe incorporation into the oxide liquid.

### 2.3 Phase Transformation

**Figure 5:**
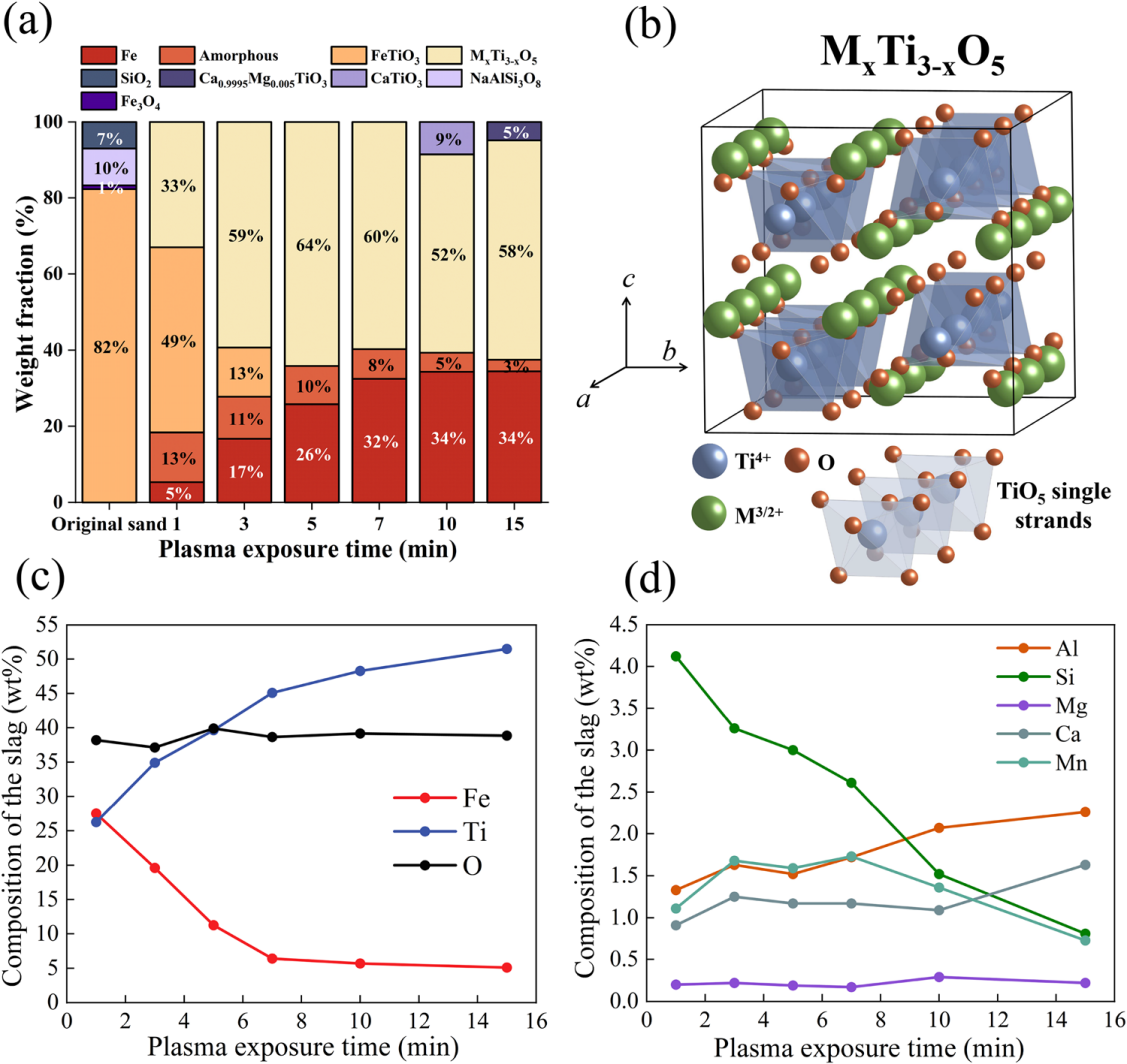
Phase transformation of LGI‐1 with hydrogen plasma processing; crystal structure of M_x_Ti_3‐x_O_5_ phase; chemical composition evolution of the titania‐rich precursor compound. a) Phase evolution of the LGI‐1 upon melting and exposure to a hydrogen‐plasma atmosphere (Ar‐10%H_2_) at different exposure times (1,3,5,7,10, and 15 min). The term M_x_Ti_3‐x_O_5_ corresponds to the sum up of all the solid solutions present in the compound that follow the M_x_Ti_3‐x_O_5_ stoichiometry. b) Crystal structure of the M_x_Ti_3‐x_O_5_ phase showing the TiO_5_ chains interconnected by M^3+^ or a mixture of M^2+^ and M^4+^ cations. c) Chemical composition evolution during reduction. Changes in weight percentage in terms of (c) Fe, Ti, and O. (d) Al, Si, Mg, Ca, and Mn.

The ilmenite concentrate samples (15 g each) were processed under a lean hydrogen‐containing plasma arc (Ar‐10%H_2_). After melting, each processed sample was allowed to solidify inside the furnace, and the metallic domains were separated from the titania‐rich material. To understand the chemistry and phase evolution during the reduction process, X‐ray diffraction (XRD) analysis of the titania‐rich compound was conducted. The XRD results are documented in **Figure** **5**a. They show that at the early stages of reduction (1 min), ilmenite contained in the LGI‐1 sample transforms into a mixture of several constituents. These are a glassy amorphous phase (13%), metallic iron (5%), and M_x_Ti_3‐x_O_5_ (0<x<2) phase (Mg_0.5_Ti_2_Fe_0.5_O_5_, 33%), and Fe_3_Ti_3_O_10_ (49%). M_x_Ti_3‐x_O_5_ (0<x<2) (where M stands for divalent and trivalent cations) consists of a complex mixture of Ti_3_O_5_, FeTi_2_O_5_, MnTi_2_O_5_, Al_2_TiO_5_, MgTi_2_O_5_, V_2_TiO_5_, and Cr_2_TiO_5_, with a pseudobrookite structure.^[^ ^13^ ^]^ Pseudobrookite crystallizes with the orthorhombic crystal structure, displaying space group D_2h_^17^‐Cmcm (63) and Z = 4 (Z = structural unit cell). In such a crystal structure, TiO_5_ octahedra are coordinated by Ti^4+^ cations, forming single‐stranded TiO_5_ chains extended along the *c*‐axis, as shown in Figure 5b. These chains are interconnected by M^3+^ or a mixture of M^2+^ and M^4+^ cations, creating a robust structural framework. The octahedral voids present between the TiO_5_​ chains exhibit significant flexibility, a fact that allows for extensive in‐phase permutations and the incorporation of various solid solution species.^[^ ^30^ ^]^

Figure 5a shows that a complete transformation of ilmenite (LGI‐1) into M_x_Ti_3‐x_O_5_ (64%) and a glassy amorphous phase (10%) and iron (26%) is achieved already after as short a time as 5 min of plasma processing. As the reduction time further increases, the titanium content within the pseudobrookite structure progressively rises as determined by EDS and depicted in Figure 5c. This fact induces a distortion of the original orthorhombic crystal structure of pseudobrookite, thus further triggering a phase transformation to a monoclinic structure comprising of Fe_0.35_Ti_2.65_O_5,_^[^ ^31^ ^]^ as observed in the specimen reduced for 15 min, in which 72% of the final oxide‐rich compound corresponds to Zr_0.03_Ti_2.66_Fe_0.05_Al_0.25_O_5_ having a monoclinic structure (Figure S1a, Supporting Information).

Since pseudobrookite cannot incorporate SiO_2_ into its structure, SiO_2_ forms a separate phase along with CaO, MnO, TiO_x_, and a portion of Al_2_O_3_, thus solidifying as a glassy amorphous phase (Figure 5a) as it has been previously reported.^[^ ^32^, ^33^ ^]^ As depicted in Figure 5a, the fraction of this amorphous phase diminishes over the course of hydrogen plasma processing so that only 3% of this phase remains in the sample processed for 15 min. This result strongly suggests that SiO‐based oxides get evaporated from the melt during the process. The presence of gangue elements such as Ca, Al, Mg, and Si plays a crucial role in the smelting process, as their distribution among various phases is influenced by the Ti^4+^/Ti^3+^ ratio.^[^ ^34^ ^]^ The continuous removal of iron from the oxide liquid (Figure 5c) results in titanium enrichment in both the pseudobrookite and within the amorphous silicate‐based phases. With the evaporation of Si (Figure 5d), the amorphous phase becomes depleted in Si and enriched in Ca, a fact that eventually leads to the precipitation of additional phases at 15 min reduction, such as CaTiO_3_ (Figure 5a)

The results shown in Figure 5a also reveal that with the precipitation of metallic iron, a significant enrichment of titanium occurs in the oxidic compound, again qualifying this process residue as a prime‐quality feedstock material for titanium and pigment production. After a maximum reduction time of 15 min, the titanium content in the oxide compound reached 51%, a value that translates into 86% “TiO_2_ equivalent” (here, “equivalent” is addressed as the titanium content independently of the state Ti^4+^/Ti^3+^). This represents a substantial increase from the initial TiO_2_ content of only 38% within the original LGI‐1. Thus, this sustainably enriched titania‐rich oxide has a high‐grade composition, rendering it a suited precursor for downstream processes, such as the sulfate method for titanium dioxide production.

Considering the capacity of HPSR to remove impurities, such as silicon (Figure 5d), from the mineral oxide mixture, a second ilmenite concentrate, characterized by higher impurity levels (LGI‐2) (as detailed in Table 1), was submitted to HPSR in order to assess the impact of gangue element concentration on the reduction process under lean hydrogen plasma conditions.

**Figure 6:**
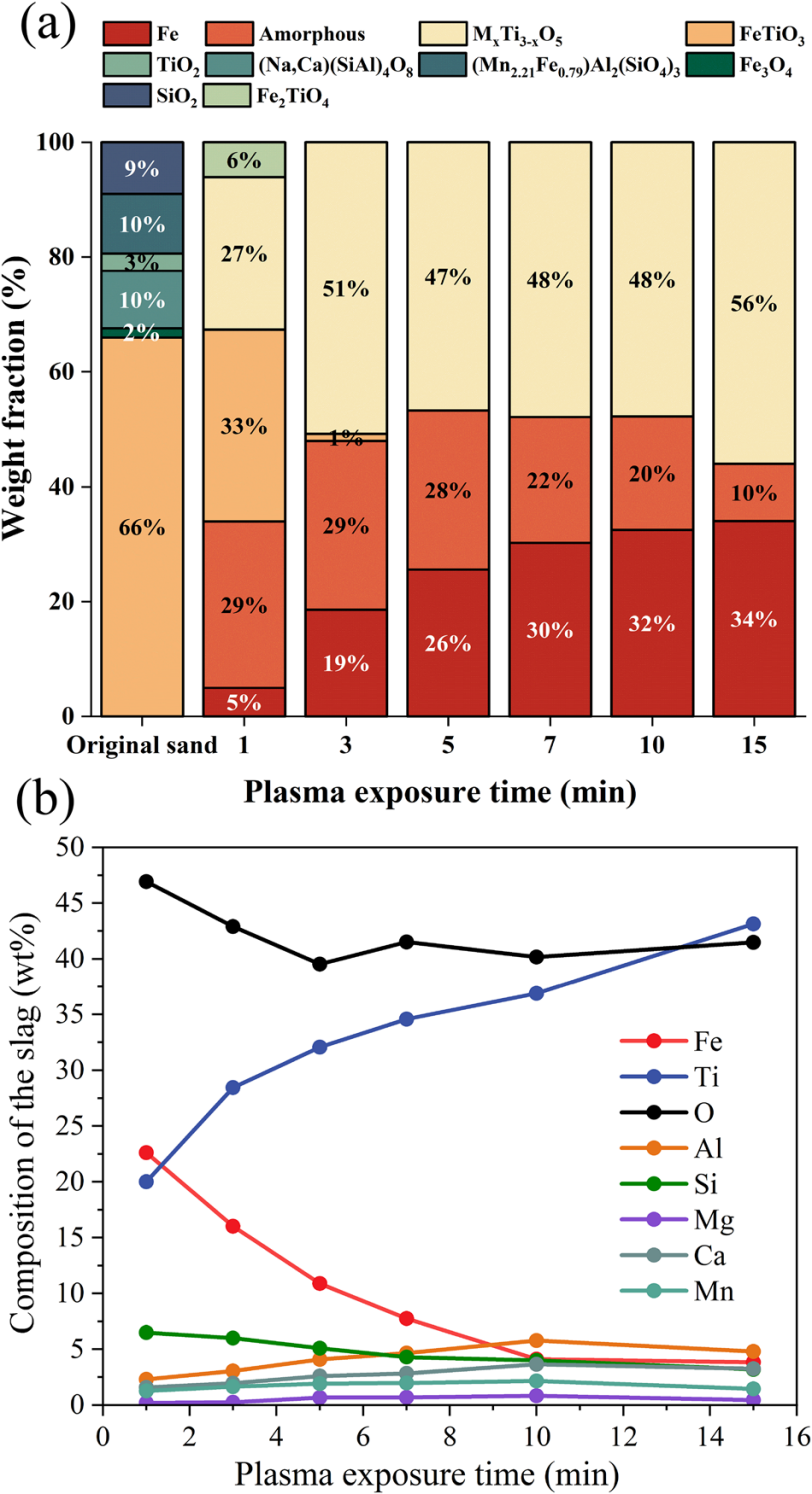
Phase transformation of LGI‐2 with hydrogen plasma processing; Chemical composition evolution during reduction. a) Phase evolution of the LGI‐2 upon melting and exposure to a hydrogen atmosphere (Ar‐10%H2) at different exposure times (1,3,5,7,10, and 15 min). The term M_x_Ti_3‐x_O_5_ corresponds to the sum up of all the solid solutions present in the titania‐rich oxide compound that follow the M_x_Ti_3‐x_O_5_ stoichiometry. b) Chemical composition evolution during reduction.

As illustrated in **Figure** **6**a, a similar transformation behavior is observed during plasma processing of LGI‐2, with a complete phase transformation into the M_x_Ti_3‐x_O_5_ phase within 5 min of plasma exposure. However, a significant proportion of the total oxide mass is composed of a silicate glassy phase, primarily attributed to the higher impurity content in the processed sample (Table 1). In particular, the concentration of SiO_2_ is approximately twice of that one found in the LGI‐1 variant, which leads to enhanced stabilization of the silicate‐based phase. This increased stabilization seems to impede the precipitation of additional phases at extended reduction times (Figure 6a. For example, at 15 min of reduction, the resulting compound is composed of 85% M_x_Ti_3‐x_O_5_ solid solution, and only 15% of the titania‐rich oxide mixture is silicate‐based phase (amorphous).

Figure 6b shows that the titanium content in the titania‐rich oxide mixture increased to 43% after 15 min reduction, corresponding to a TiO_2_ equivalent concentration of 72%, again a most significant improvement from the initial 29% TiO_2_, thus turning a mixed mineral waste product into valuable feedstock for the titanium industry. Furthermore, the initial SiO_2_ concentration, which was 19%, was reduced to 7%. This substantial decrease in SiO_2_ content underscores the cleaning effect of HPSR,^[^ ^17^, ^18^, ^35^ ^]^ confirming its efficiency in enhancing the purity of the resultant titania‐rich precursor for subsequent applications. This enhanced purity is critical for subsequent processing steps, such as the sulfate process, where elevated silicon dioxide (SiO_2_) levels, typically ranging between 17–20 wt.%, inhibit acidolysis and adversely affect the efficiency of TiO_2_ production.^[^ ^36^ ^]^

### 2.4 Mechanisms Behind the Chemical Transformations

A thermodynamic analysis of the ilmenite reduction process was conducted by simulating the exposure of a 15‐g sample of molten LGI‐1, to incrementally increasing amounts of 90%Ar‐10%H_2_ at a temperature of 1600°C (see Experimental Section). At this temperature, ilmenite (FeO.TiO_2_) melts, thus its crystal structure breaks down into the corresponding ionic and neutral components such as Fe^2+^, Al^3+^, Mg^2+^, Ca^2+^, Mn^2+^, SiO_4_^4−^, O^2−^, TiO_2_, and AlO_2_^1−^.

**Figure 7:**
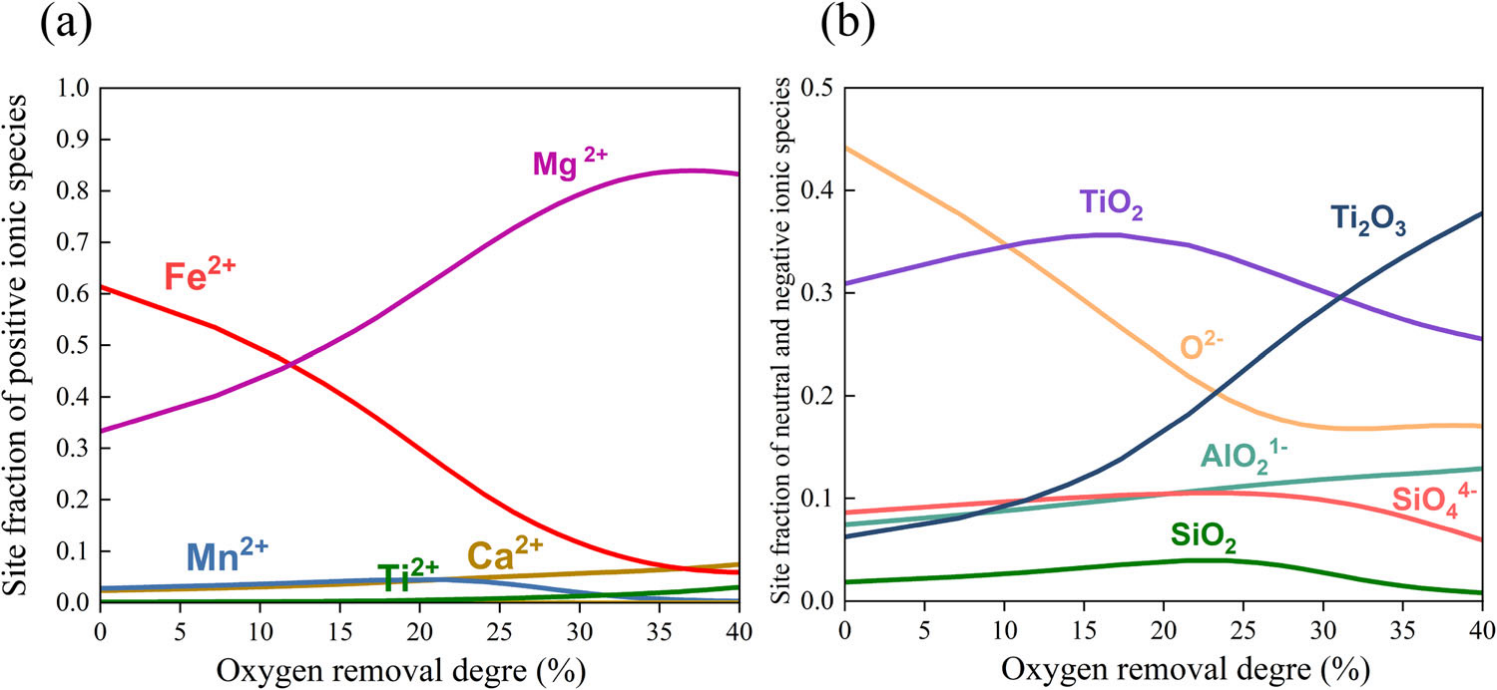
Results of thermodynamic calculations of LGI‐1 exposed to Ar‐10%H_2_ gas mixture at 1600 °C. a) Site fraction of the positive ionic species. b) Site fraction of the negative ionic species.

The reduction process was examined through a detailed analysis of the site fractions of key ionic and oxide species within the liquid phase throughout the progression of the reaction (i.e., oxygen removal), as depicted in **Figure** **7**a. This figure shows a rapid decline in the site fractions of Fe^2+^ and O^2−^, indicating that the continuous oxygen removal promotes the reduction of Fe^+2^ ions into metallic Fe liquid. When hydrogen plasma species interact with the molten material, redox reactions take place at the interface between the plasma arc and the melt's surface. The reduction is driven by the temperature‐dependent free energy of formation for different oxides. Iron, which has the lowest affinity for oxygen among the metals contained in ilmenite, is reduced first through reactions such as FeO + 2H^+^ +2e^−^ → Fe + H_2_O. Concurrently, Figure 7b shows a slight decrease in the TiO_2_ site fraction from 15% reduction onward, alongside a corresponding increase in the concentration of Ti_2_O_3_ species, clearly evidencing the partial reduction of TiO_2_ into lower oxides (Ti_2_O_3_). Thus, it is expected that a partial reduction of titanium dioxide (TiO_2_) also occurs through the following reaction: 2TiO_2_ + 2H^+^ +2e^−^ → Ti_2_O_3_+ H_2_O. Upon cooling, the formed Ti_2_O_3_ is incorporated into the pseudobrookite structure following the M_x_Ti_3‐x_O_5_ stoichiometry (as shown in Figures 4 and 5). XRD analysis confirms that overreduction of TiO_2_ is effectively avoided within the applied reduction timeframes, as neither Ti metal nor Ti_2_O_3_ is observed to precipitate as a discrete phase. This outcome is advantageous since Ti_2_O_3_ exhibits significantly lower solubility in sulfuric acid, rendering it less suitable for processes involving the sulfate route.^[^ ^37^, ^38^, ^39^ ^]^

The observed decrease in SiO_2_ content at 25% reduction (Figure 7b) provides evidence for the evaporation of SiO_2_ as suboxide species (SiO). Furthermore, the gas‐phase site fractions presented in Figure S3 (Supporting Information) corroborate the presence of SiO, thereby supporting the proposed mechanism.

### 2.5 Chemical Partitioning and Microstructural Evolution

#### 2.5.1 Low Grade Ilmenite‐1

**Figure 8:**
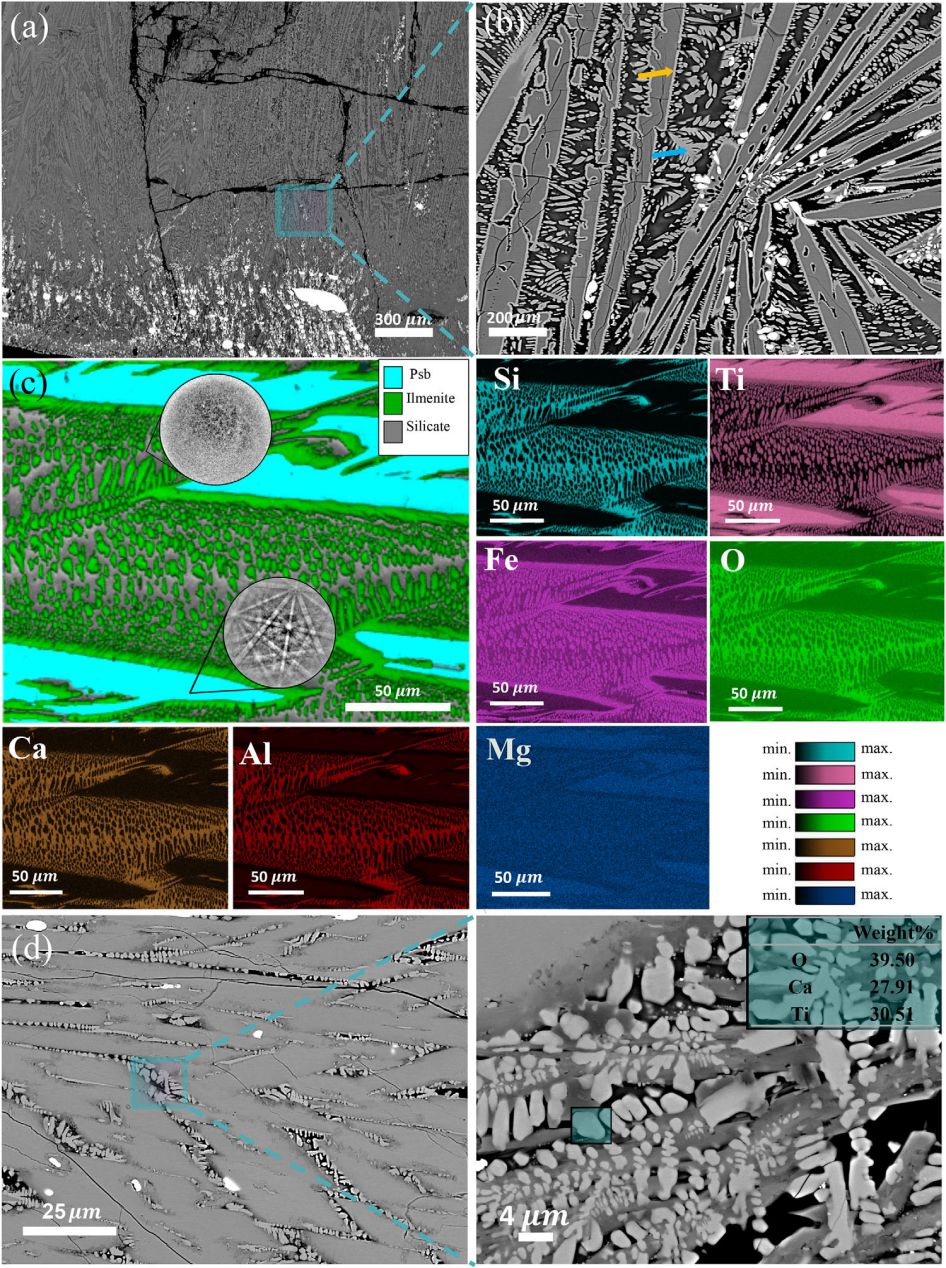
Microstructure and local chemical composition of the LGI‐1 processed at different reduction times. (a) BSE‐SEM image of the cross section of the sample processed for 3 min, iron nodules sit at the bottom of the sample due to the density difference. b) Enlarged view of the image a) depicting the existence of different phases in the solidified titania‐rich compound, pseudobrookite, ilmenite, silicate, and iron. The yellow arrows indicate the precipitation of ilmenite along the pseudobrookite plates, and the blue arrows point to the precipitation of ilmenite as dendrites. c) EBSD phase map of the sample processed for 3 min showing pseudobrookite, ilmenite, and silicate phase in blue, green, and grey, respectively, as an inset the Kikuchi pattern obtained from the Si‐enriched phase and the pseudobrookite phase are shown. The elemental maps show the chemical partitioning of elements into the different phases. d) Microstructure of the sample processed for 15 min, highlighting the presence of CaTiO_3_.

The local chemical partitioning during the reduction process was investigated by analyzing the solidified microstructure of the sample processed for 3 min using scanning electron microscopy (SEM) coupled with energy‐dispersive X‐ray spectroscopy (EDS) and electron backscatter diffraction (EBSD). In the early stages of reduction for the LGI‐1 sample (3 min), splatter‐like iron formations were observed dispersed across the top surface of the sample. Additionally, iron nodules accumulated predominantly at the bottom regions of the sample, likely a result of their higher mass density relative to the remaining oxide mixture, thus causing them to sink, as illustrated in the SEM‐BSE images in **Figure** **8**a,b.

The SEM‐EDS analysis of the cross‐section of the partially‐reduced Titania‐enriched oxide compound obtained after freezing the sample by rapid cooling via the copper hearth reveals that it primarily solidifies as three distinct phases, as depicted in the phase map shown in Figure 8c. The microstructure reveals a plate‐like pseudobrookite phase, which grows within the darker, glassy silicate phase. This microstructural evolution is driven by the disparity in melting points of the constituent phases. Upon initiation of rapid solidification, imposed by the water‐cooled crucible hearth, the high‐melting point pseudobrookite phase (melting point ≈1600°C) begins to precipitate first, forming the characteristic needle and plate‐like structures (Figure 8b). Once the pseudobrookite phase has completely solidified, ilmenite, which has a melting point between 1300 and 1500 °C, commences to solidify. This phase precipitates along the pre‐existing pseudobrookite plates (as highlighted by the yellow arrows in Figure 8b). Due to the rapid cooling rate, the equilibrium partitioning of ilmenite is hindered, leading to its dendritic precipitation within the remaining silicate melt (as indicated by the arrows in blue in Figure 8b). The final phase to solidify comprises silicate‐based compounds, which occupy the regions between the pseudobrookite and ilmenite phases. This phase, with a melting point in the range of 1100–1300 °C,^[^ ^40^, ^41^ ^]^ forms as the residual liquid solidifies, filling the space between the previously crystallized phases, as illustrated in the dark regions of the micrograph shown in Figure 8b. This sequential solidification process, driven by differential phase stabilities and rapid cooling, results in a heterogeneous microstructure where the high‐melting pseudobrookite phase serves as the structural backbone, decorated by ilmenite dendrites, all within a glassy silicate matrix.

Elemental mapping of the titania‐rich oxide mixture cross‐section (Figure 8c) reveals distinct compositional partitioning between the pseudobrookite plates, the ilmenite, and the surrounding glassy phase. The pseudobrookite plates exhibit significant enrichment in titanium, while being notably depleted in Si, Ca, and Al. This distribution suggests the preferential partitioning of titanium into the pseudobrookite phase during the early stages of plasma exposure. In contrast, the surrounding glassy phase is enriched in Ca, Si, O, and Al, forming a silicate‐based matrix. The amorphous nature of this phase was further confirmed by the inspection of the Kikuchi pattern captured during EBSD analysis of the LGI‐1 and documented in Figure 8c. The Kikuchi pattern obtained from the Si‐enriched phase (Figure 8c) lacks the crystalline diffraction features typically observed in well‐ordered structures, confirming the formation of a disordered glass‐like compound.^[^ ^42^ ^]^

Upon complete transformation of ilmenite into the M_x_Ti_3‐x_O_5_ phase, prolonged plasma exposure leads to the partial evaporation of silicon in the form of SiO, accompanied by an enrichment of Ca and Ti in the silicate matrix (See section “Mechanism behind the chemical transformations”). This compositional shift promotes the precipitation of additional phases, notably the formation of dendritic CaTiO_3_ structures. These dendrites emerge within the interstitial regions between the large pseudobrookite plates and the residual silicon‐rich phase (Figure 8d).

To confirm the observed partial evaporation of Si from the oxide melt, a Cu cylinder, also referred to as ‘cold finger’, was installed on the anode of the furnace to serve as an electrostatic filter, thus capturing evaporated substances for posterior analysis (see more details in Experimental Section). SEM‐EDAX analysis of the cold finger's surface, extracted from the furnace after 10 min of experiments, confirmed the evaporation Si together with minor amounts of Mn and Fe as shown in Figure S2a (Supporting Information). Thermodynamic calculations were also conducted to identify the elements present in the gas phase during the reduction process, and the obtained results are shown in Figure S2b (Supporting Information). These calculations confirmed the evaporation of the same elements that were detected on the surface of the cold finger.

#### 2.5.2 Low Grade Ilmenite‐2

**Figure 9:**
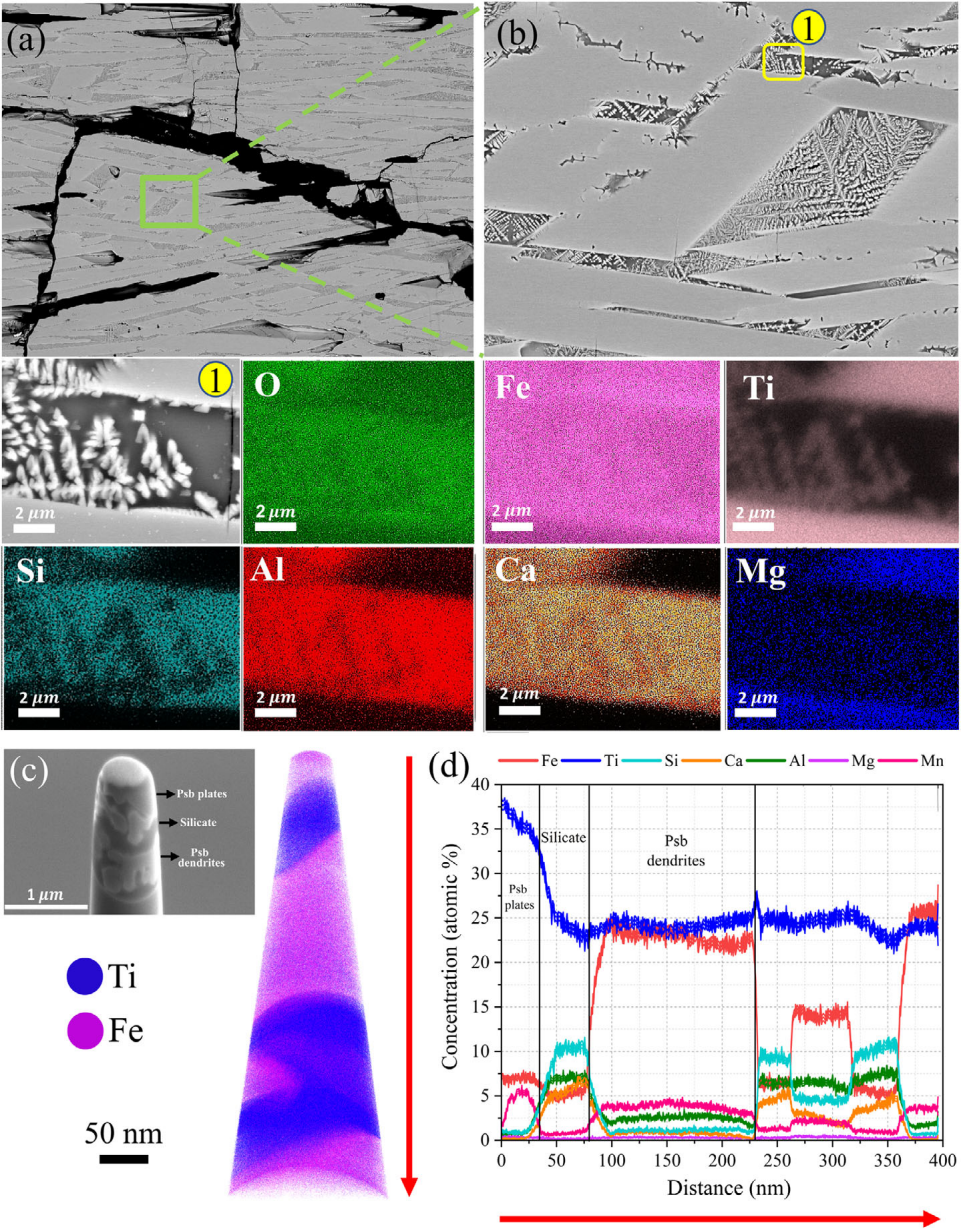
Microstructure and local chemical composition of LGI‐2 processed for 15 min; Atom probe tomography (APT) analysis of the interface between pseudobrookite plates, pseudobrookite dendrites, and silicate phase in the partially reduced sample for 15 min. a) Microstructure of sample reduced for 15 min. b) enlarged view highlighting the presence of pseudobrookite plates and pseudobrookite dendrites; elemental distribution map. c) APT tip before the final annular milling step, 3D atom probe tomography maps of Fe and Ti; “psb” refers to pseudobrookite. d) Concentration profiles of Fe, Ti, Si, Ca, Al, Mg, and Mn relative to the position of the interfaces.

For the LGI‐2, a comparable microstructure is observed during the initial stages of reduction (e.g., 1, 3, and 5 min). However, at extended reduction times (15 min), the microstructure evolves to display a different morphology, with large pseudobrookite plates, regions of silicate phase, and pseudobrookite dendrites embedded within the silicate matrix, as shown in **Figure** **9**a,b. Both the dendrites and plates are pseudobrookite solid‐solutions, exhibiting slight compositional variations. As demonstrated in the corresponding elemental maps, the dendritic structures are enriched in iron relative to the pseudobrookite plates. This compositional differentiation indicates localized partitioning during solidification.

The exact compositional differences between the dendritic pseudobrookite and the plate‐like pseudobrookite could not be fully resolved using SEM‐EDS due to the fine scale of the dendritic phases, which fall below the resolution limit of the technique. To elucidate the compositional variations within these nano‐sized dendritic pseudobrookite phases, atom probe tomography (APT) was employed. A specimen was extracted from the interface between the pseudobrookite dendrites, pseudobrookite plates, and the surrounding silicate phase, as illustrated in Figure 9c. In this figure, the upper region corresponds to the plate‐like pseudobrookite, while the dendritic pseudobrookite appears as whiter phases embedded within the dark silicate matrix.

The compositional profile of the APT specimen along the longitudinal direction (from top to bottom, as indicated by the arrow in Figure 9c) is presented in Figure 9d. This analysis reveals that the pseudobrookite plates are enriched in Ti, with a concentration of ≈39 at.% (indicated by the blue line), while being comparatively depleted in Fe at ≈7 at.% (red line in Figure 9d). As the profile moves toward the silicate‐rich region, the titanium concentration decreases to ≈23 at.% and the Si concentration increases to ≈10 at.%. In contrast, the dendritic pseudobrookite phase exhibits a titanium concentration similar to that of the adjacent silicate phase, but with a significantly higher Fe concentration of ≈23 at.%.

Notably, compositional variations are evident within the dendritic pseudobrookite. While the titanium concentration remains relatively constant, the Fe content fluctuates, particularly in the region from 240 to 400 nm, as shown in the compositional profile. This analysis indicates that although titanium preferentially partitions into the pseudobrookite phases (both dendrites and plates), the resulting compositions are not uniform. Instead, variations exist both between the plate‐like and dendritic pseudobrookite phases and within different regions of the dendrites.

These findings suggest that a highly enriched titania precursor is, in fact, a complex mixture of solid solutions of various Ti‐enriched phases (M_x_Ti_3‐x_O_5_) that share a similar crystal structure as it has been previously reported.^[^ ^39^, ^43^, ^44^ ^]^ This structural similarity results in the phases solidifying together, appearing as a single phase when examined by SEM‐EDS. However, the XRD analysis suggests the coexistence of multiple M_x_Ti_3‐x_O_5_ type solid solutions phases (Mg_0.5_Ti_2_Fe_0.5_O_5_, Fe_3_Ti_3_O_10_, Mg_0.3_Ti_2.7_O_5_, FeTi_2_O_5_) as detailed in Figure S1 (Supporting Information). The presence of compositional variations at the nanometer scale, as revealed by APT, supports this interpretation that the titania compound is a mixture of different solid solutions rather than a homogeneous phase. From a practical standpoint, the observed chemical inhomogeneities within the Ti‐rich precursor do not pose significant challenges to its application or utility in further processing, such as the sulphate process for TiO_2_ production

From an energy consumption and CO_2_ generation perspective, detailed calculations (Notes S1–S3, Supporting Information) show that producing one ton of Titania‐rich compound via hydrogen plasma smelting reduction requires **2177.5 kWh**, compared with **2668 kWh** for the conventional carbothermic route.^[^ ^45^ ^]^ This corresponds to an **18.4% reduction in energy demand**. Beyond the direct energy savings, HPSR yields a refined grade of metallic iron that can be directly used in steelmaking, thereby eliminating the need for post‐processing. By contrast, carbon‐based reduction routes introduce impurities such as S, P, and Si, which necessitate additional refining steps that are energy intensive. While the precise energy savings associated with bypassing conventional refining are challenging to quantify and are therefore not reported here, the avoidance of these processes represents a clear additional reduction in energy consumption. Further energy costs that might have to be added on the carbothermic side are CO_2_ capture, valorisation, and/or storage, all aspects emerging today in current legislative measures as additional constraints.

The CO_2_ emissions associated with the carbothermic reduction of ilmenite for the production of Titania‐rich compounds stem predominantly from two sources: ≈82% from electricity consumption and 18% from carbon‐based reduction reactions.^[^ ^15^ ^]^ Implementation of the hydrogen plasma smelting reduction (HPSR) process enables an 18.4% reduction in energy demand, corresponding to a 15% decrease in CO_2_ emissions attributable to electricity use. In parallel, replacing carbon with green hydrogen as the reductant fully abates 18% of emissions previously associated with the chemical reduction step. Together, these modifications result in a 34% net reduction in total CO_2_ emissions, comprising a 15% contribution from improved energy efficiency and a 19% contribution from the complete decarbonization of the reductant.

However, the proposed pathway for the production of Titania‐rich compounds and high‐purity iron through HPSR assumes complete reliance on renewable electricity, with carbon reductants replaced entirely by green hydrogen. By using this approach, we are able to quantify the CO_2_ emissions of our process as nearly zero.

## 3 Conclusion

Here, we have demonstrated that by using hydrogen plasma smelting reduction, ilmenite concentrates of varying composition can be processed in a sustainable way. The process proceeds with fast kinetics and transforms low‐grade concentrates into valuable products, such as a high‐titania containing oxide compound and metallic Fe, while producing only water as a by‐product. By achieving rapid reduction kinetics, the process enables the selective recovery of high‐purity metallic Fe and the formation of titania‐rich oxide compounds with TiO_2_ equivalents of up to 86%, suitable as precursor feedstock for pigment‐grade TiO_2_ production via the sulfate route. The elimination of direct CO_2_ emissions, coupled with efficient impurity removal, including significant reductions in SiO_2_ content, and the avoidance of TiO_2_ reduction to less soluble phases, underscores the process's environmental and technical advantages over conventional carbothermic and synthetic rutile methods. The demonstrated flexibility of HPSR to process ilmenite feedstocks of varying quality, alongside its dual product output, positions it as a technology for sustainable titanium and iron production in a resource‐constrained and climate‐conscious future.

## 4 Experimental Section

### Material

In this study, two distinct ilmenite concentrates, each differing in chemical composition, were utilized. The chemical compositions of these concentrates are presented in Table 1.

The chemical composition of the concentrates was determined using XRF, while phase characterization was carried out via XRD. The morphology and particle size distribution were analyzed through SEM‐EDS.

### Reduction Experiments

The ilmenite concentrates were compacted into pellets of ≈15 g under a pressure of 20 MPa. Samples were placed on the water‐cooled copper hearth of an electric arc melting furnace. The furnace was equipped with a tungsten electrode (6.34 mm in diameter), and for the experiments, its chamber was filled with a static gas mixture of Ar–10%H_2_ at an absolute pressure of 900 mbar (i.e., no continuous gas flow was applied). To simultaneously melt and reduce the samples, a plasma arc was ignited between the electrode tip and the input material using a current of 200 A and a voltage of 24 V, corresponding to a power input of 4.8 kW.

The reduction process was conducted through a series of sequential melting runs. Initially, samples were melted in a 100% Ar atmosphere for 2 min. Subsequently, they were exposed to the reducing atmosphere (Ar‐10% H_2_ gas) for a defined number of cycles (1, 3, 5, 7, 10, and 15 cycles), with each cycle corresponding to a 1‐min exposure to the hydrogen plasma. For each exposure, three samples were processed and analyzed.

### Phase Quantification

After solidification of the samples, they were crushed to separate the reduced iron from the remaining titania‐rich compound. Both iron and the titania‐rich oxide portions were weighed using a precision analytical balance (with an accuracy of ± 0.0001 g). The titania‐rich compound was further ground down to 90 µm and probed by X‐ray diffraction (XRD) using a diffractometer D8 Advance A25‐X1, equipped with a cobalt Kα X‐ray source, operated at 35 kV, 40 mA. The resulting diffraction data were processed using the Brucker Topas v. 5.0 software. Rietveld refinement was utilized to ascertain the weight fraction of the constituents in the powder. The refinement of peak identification was carried out using analysis software with a mineral database (MDI JADE 9.0). To evaluate the kinetics of iron reduction, the weight of the sub‐micron sized iron domains entrapped within the oxide portion was combined with that of the mm‐sized iron extracted from the same sample.

### Microstructural Characterization

The solidified samples were cut and embedded in resin for conventional metallographic preparation. The microstructural characterization was performed using a Zeiss Merlin scanning electron microscope (SEM). Complementary energy‐dispersive X‐ray spectroscopy (EDS) and electron backscatter diffraction (EBSD) measurements operating at 15 KV and 200nA were also performed to investigate the local chemistry and phase distribution of the partially reduced samples.

### Thermodynamic calculations

Thermodynamic calculations were carried out using the Thermo‐Calc program in conjunction with the SSUB5 SGTE (Scientific Group Thermodata Europe) Substances Database for the description of the gas phase and the TCS Metal Oxide Solutions Database (TCOX10) for the description of the liquid phases. The initial condition for the calculations was 15 grams of ilmenite concentrate composition, which was maintained at 1600°C while increasing amounts of a gas mixture of Ar‐10%H_2_ were added concurrently. Throughout the simulations, element partitioning across all constituents was allowed. The total pressure was 1 × 10^5^ Pa.

The liquid phase was represented using a two‐sublattice model, incorporating the following species: (Fe^2+^_,_ Mn^2+^, Ti^2+^, Ca^2+^, Si^4+^, Al^3+^, Mg^2+^)_1_(O^2−^, SiO_4_^4−^, VA^−^, FeO_1.5_, TiO_1.5_, MnO_1.5_, TiO_2_, AlO_2_^1‐^, SiO_2_)_1._ To maintain charge neutrality, the concentration of charged vacancies adjusts according to the composition. The specific position occupied by each species within the sublattice was referred to as its “site fraction.”

### Atom Probe Tomography

Atom probe tomography (APT) was employed to characterize the atomic‐scale elemental distribution across the hetero‐interfaces between the pseudobrookite plates, pseudobrookite dendrites, and silicate phases existing in the sample reduced for 15 min. The APT specimens were prepared using the site‐specific liftout method using a FEI Helios NanoLab600i dual‐beam focused ion beam (FIB) SEM. The final annular cleaning process of the tips was performed at 5 kV and 16 pA, to minimize beam damage. The APT measurements were conducted using a Local Electrode Atom Probe (LEAP, Cameca Instruments Inc.) 5000 XS with a detection rate of 0.7%. The instrument was operated at a 200 kHz laser pulse frequency and laser energy of 60 pJ. The base temperature was maintained at 50 K during the measurements. The dataset was reconstructed using a standard voltage protocol in the commercial software AP Suite 6.3.

### Electrostatic Filter

A thin copper (Cu) cylinder, referred to as “cold‐finger,” was installed at the electrical contact of the anode. This setup allowed the cold finger to function as an electrostatic filter, attracting specific species present in the furnace atmosphere. After completing the experiments, the cold‐finger was removed from the furnace and preserved for subsequent analysis. Local chemical composition was examined using scanning electron microscopy (SEM) combined with energy dispersive X‐ray spectroscopy (EDS). To prevent charging effects during these analyses, the cold‐fingers were coated with a thin (∼9 nm) carbon layer.

## Conflict of Interest

The authors declare no conflict of interest.

## Supporting information



Supporting Information

## Data Availability

The data that support the findings of this study are available from the corresponding author upon reasonable request.
