# Supplementary material for: Toward Decarbonization of the Titanium Industry via Hydrogen Plasma Smelting Reduction
Source: Adv Sci (Weinh). 2025 Oct 20;13(1):e14689. doi: 10.1002/advs.202514689 (PMC12766993; doi:10.1002/advs.202514689)
Supplement: Supplementary file 1 — Supporting Information [file ADVS-13-e14689-s001.docx]

Supporting Information

**Towards decarbonization of the titanium industry via hydrogen plasma smelting reduction**

*Laura Gabriela Torres-Mejia^1*^, Ubaid Manzoor^1^, Guangyi Guo^1^, Chris W. Bumby^2,3^, Dierk Raabe^1^, Isnaldi R. Souza Filho^1,4*^.*

**Figure S1. Phase evolution of the ilmenite concentrates (LGI-1 and LGI-2) upon exposure to hydrogen plasma smelting reduction. (a)** Phase evolution for the LGI-1 upon melting and exposure to a hydrogen atmosphere (Ar-10%H_2_) at different exposure times (1,3,5,7,10 and 15 min). presence of multiple solid solutions with M_x_Ti_3-x_O_5_ stoichiometry which form a mixture corresponding to the final titania-rich compound. **(b)** Phase evolution for the LGI-2 upon melting and exposure to a hydrogen atmosphere (Ar-10%H_2_) at different exposure times (1,3,5,7,10 and 15 min). presence of multiple solid solutions with M_x_Ti_3-x_O_5_ stoichiometry which form a mixture corresponding to the final titania-rich slag compound.

**
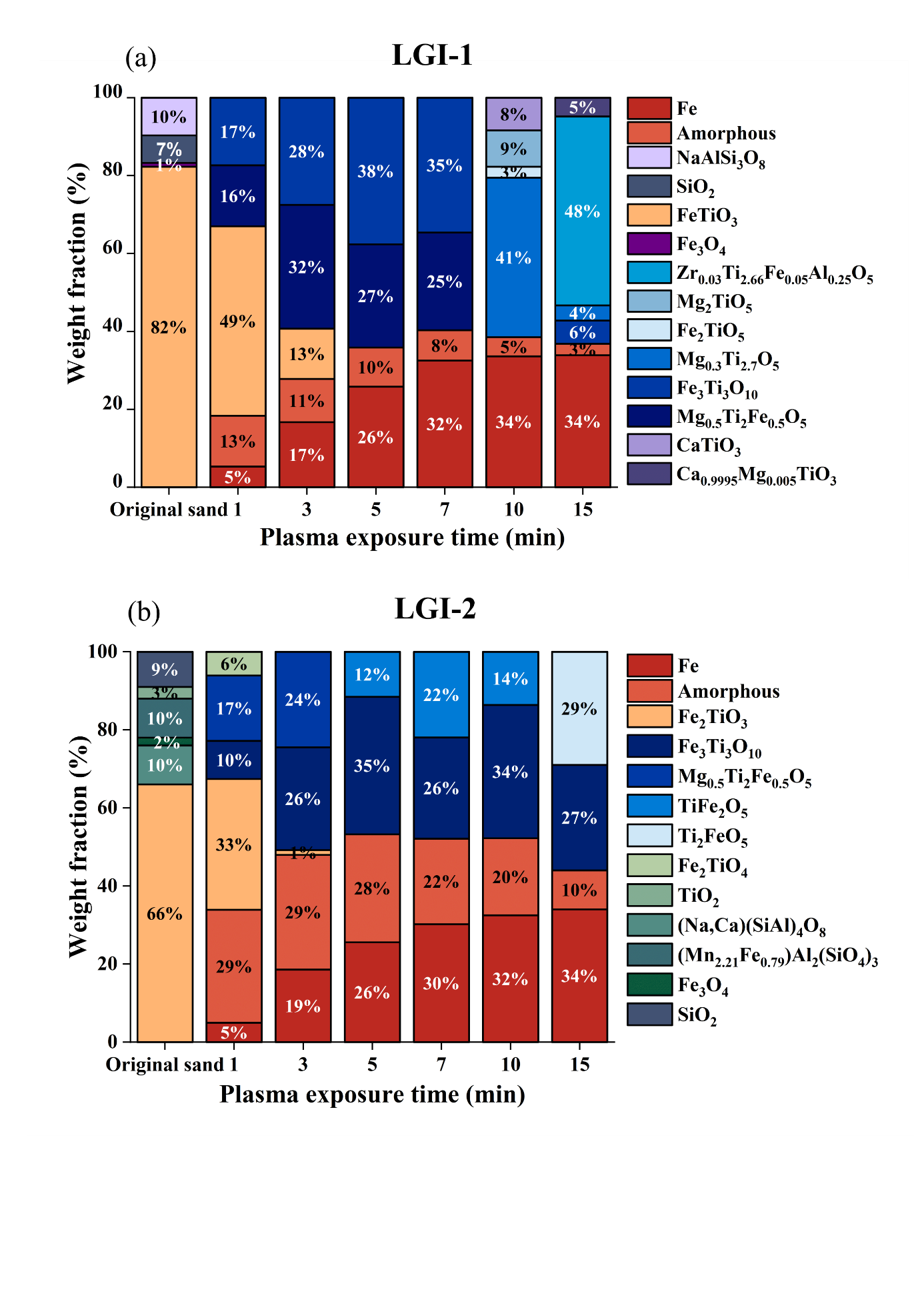
**

**Figure S2. Cold finger analysis. (a)** SEM-EDS analysis of the cold finger after 10 min of exposure time to hydrogen plasma. **(b)** Thermodynamic calculation of the elements present is the gas phase as a function of the oxygen removal degree (wt%).


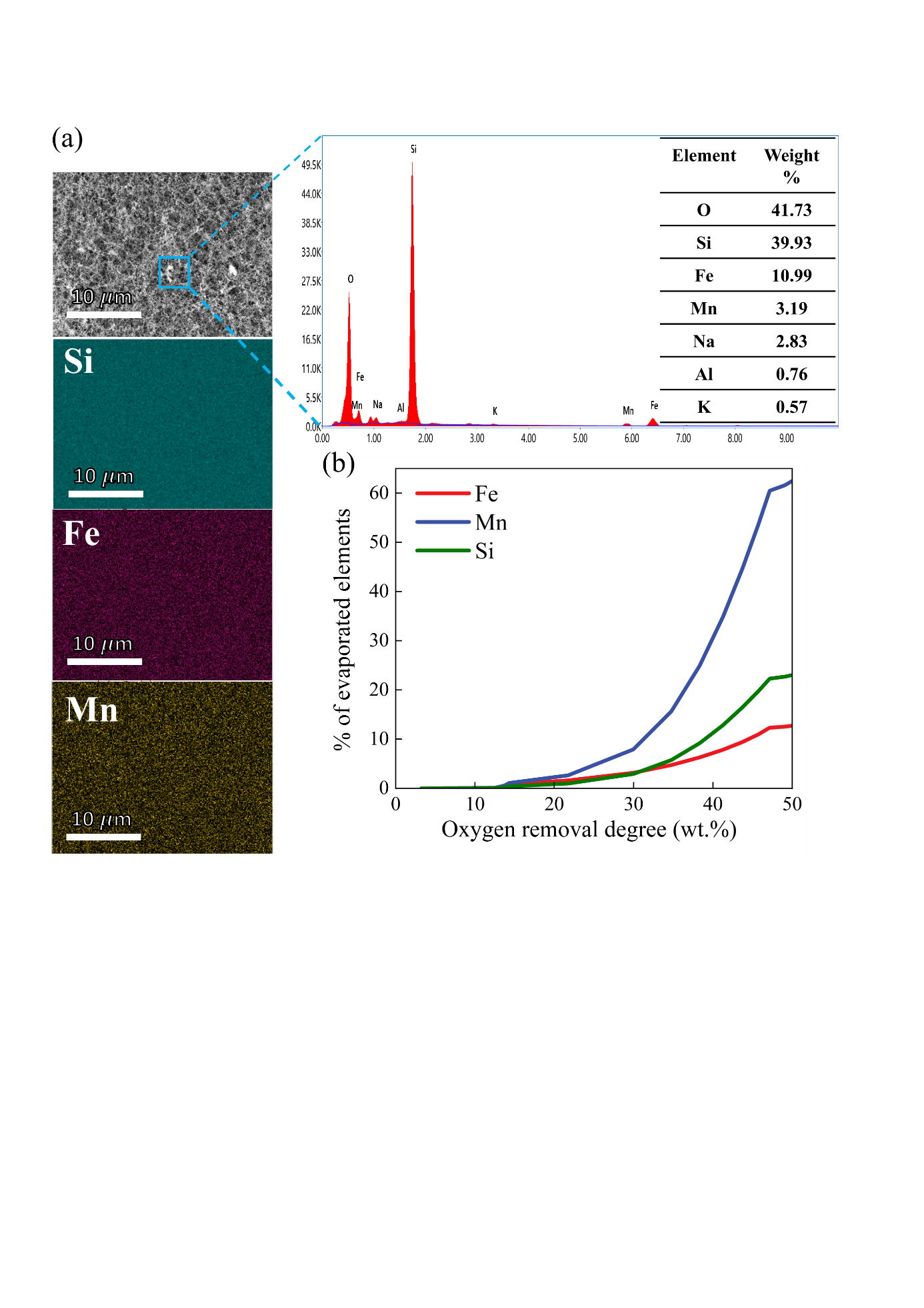


**Figure S3. Site fraction of constituents in gas phase.** Constituents of the gas phase of LGI-1 exposed to Ar-10%H_2_ gas mixture at 1600°C.


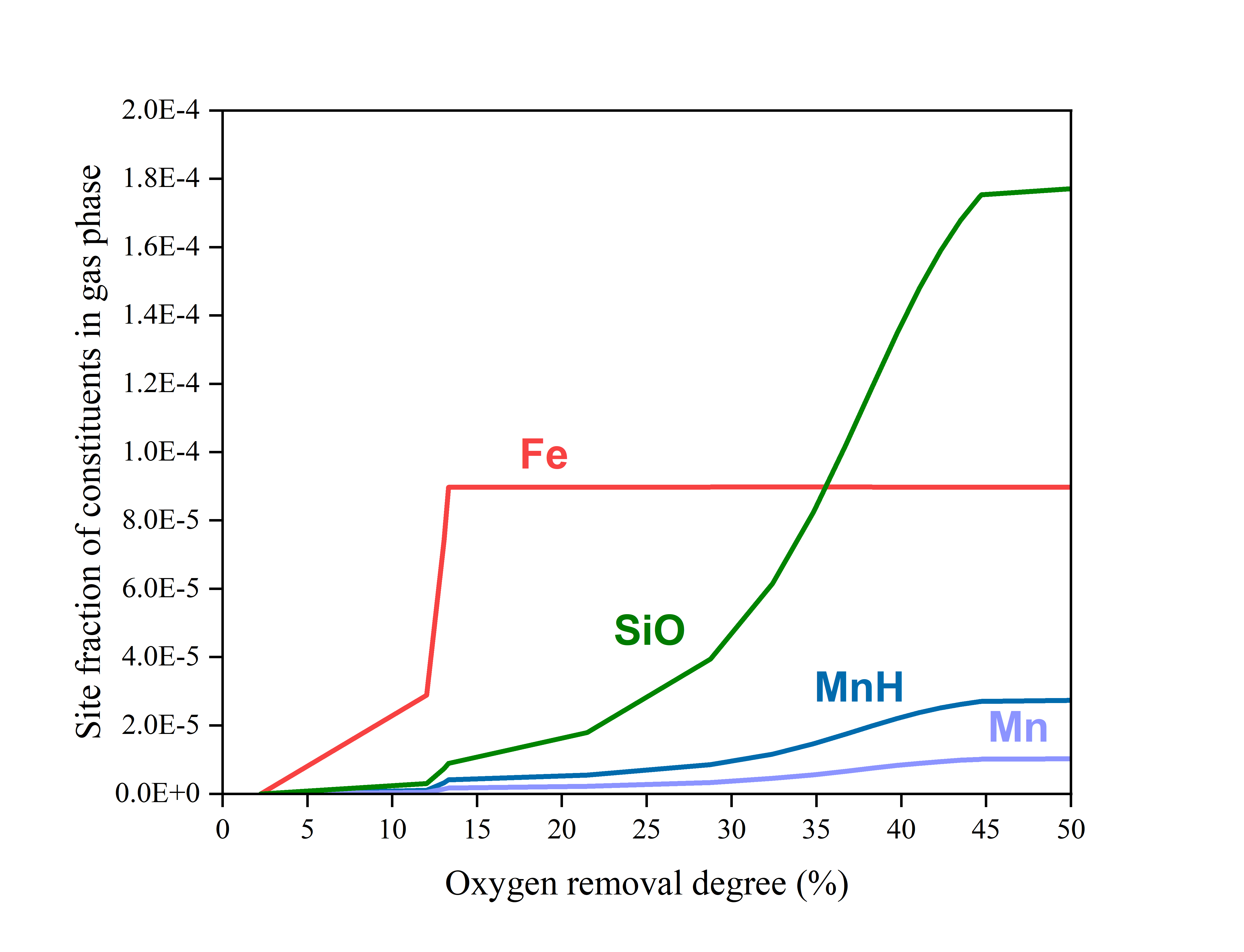


**Supplementary Notes**

**Supplementary Note 1.** **Theoretical estimations of hydrogen requirement:**

Basis: 1 tone of Titania-rich compound produced from 1.82 ton of LGI-2 concentrate

**Reduction of Fe oxide to Fe**

(Fe^+2^+O^-2^) +2H = Fe +H_2_O

The maximum recovery as shown in the main file was 93% for

When producing 1 ton of Titania-rich compound 442 kg of Fe are produced

Amount of Fe recovered= 442 kg

*Oxygen removed to produce 442 kg of Fe*

Moles of Fe=442 kg Fe/58.69 kg/ mol =7531.09 mol Fe

Mass of oxygen removed =7531.09 mol O * 16 g/mol = **120.49 kg**

**Partial reduction of TiO_2_ to Ti_2_O_3_**

2TiO_2_ + 2H = Ti_2_O_3_ + H_2_O

The composition of the Titania-rich compound follows a relationship of the equivalent FeO to equivalent Ti_2_O_3_ in the final product, where: ^[1]^

(%FeO)eq = (%FeO) + (MFeO/MMgO)(%MgO) + (MFeO/MMnO)(%MnO)

Similarly, the equivalent Ti_2_O_3_ content is calculated as:

(%Ti_2_O_3_)eq = (%Ti_2_O_3_) + (M Ti_2_O_3_ / MV_2_O_5_ )(%V_2_O_5_) + (M Ti_2_O_3_ / MCr_2_O_3_ )(%Cr_2_O3) + (M Ti_2_O_3_ / MAl_2_O_3_ )[(%Al_2_O_3_) – (%SiO_2_)/3]

In this case the TiO_2_ equivalent corresponds to 72% and the TiO2 content corresponds to 52.9% and Ti_2_O_3_ content to 19.1%

Therefore, for 1 ton of titania-rich compound, we have the following results:

TiO_2_equivalent: 720 kg

TiO_2_ content: 529 kg

Ti_2_O_3_ content: 191 kg

Moles of Ti_2_O_3_ = 191 kg Ti_2_O_3_ /143.76 g/mol = 1328.6 mol of Ti_2_O_3_

Mass of oxygen removed = 1328.6 mol of Ti_2_O_3_ *16 g/mol= **21.257 kg**

Total oxygen removed to produce 1 ton of titania rich compound + 442 Kg of Fe = **141.747 kg**

Hydrogen Requirement

For the removal of 1 mol of O, 2 mol of H are required

Total moles of O removed = 141.747 kg/16 g mol = 8859.18 mol O

Hydrogen required to produce 1 ton of Titania-rich compound = 8859.18 mol O * 2 g/mol H_2_ = 17719.37 g H_2_ = **17.71 kg H_2_**

**Supplementary Note 2.** **Energy requirement**

The fundamental difference between the current and proposed processes lies in the replacement of carbon-based reductants and conventional energy sources with greener alternatives, such as renewable hydrogen and clean or green electricity. The proposed process provides a fully electrified pathway for the production of titania-rich compounds and ready-to-use iron.

The thermodynamic energy requirements to heat and melt the ore remain largely similar in both processes. The primary difference in energy demand arises from the reaction enthalpies associated with the different reductants. We provide detailed calculations comparing the reaction enthalpies, which quantify the energy required for the chemical reactions in these two methods.

**Energy required for heating and melting**

In the carbothermic reduction route, the energy demand for smelting ilmenite in a 30 MVA direct current electric arc furnace has been reported as approximately **1003.31 kWh per ton of ilmenite**, with additional system heat losses of **996.69 kWh per ton of ilmenite.** ^[2]^

For the production of **1 ton of Titania-rich compound**, the total energy requirement corresponds to **2668 kWh**, distributed as follows:

- **Energy for reduction:** 1334 kWh
- **Energy consumed by reactions:** 444.6 kWh (approximately one third of the total ^[3]^)
- **Heat losses:** 1334 kWh

Therefore, the energy required for heating and melting the ilmenite sand corresponds to **889.4 kWh per ton of Titania-rich compound.**

**Energy required for the reaction**

The energy consumed by chemical reactions (Eᵣ) was calculated using thermodynamic data from the JANAF tables. ^[4]^ The reactions were assumed to occur in two sequential steps.

1. Conversion of molecular H_2_ into plasma species:
2. Reduction reaction between metal oxides and plasma species:

The net enthalpy change of the reaction is

𝛥𝐻𝑟 = 𝛥𝐻_1_ + 𝛥𝐻_2_

Where:

*ΔH_r_*​=Net enthalpy change of reaction.

*ΔH_1_​*=The enthalpy change associated to the conversion of molecular H_2_ into plasma species

H_2_ = 2H/H^+^/H^*^, (ΔH_1_)

*ΔH_2_*​= The enthalpy change associated to the reaction between metal oxides and plasma species:

Fe^2+^ + O^2-^ + 2H/H^+^/H^*^ = M + H2O, (ΔH_2_)

2TiO_2_ + 2H/H^+^/H^*^ = Ti_2_O_3_ + H_2_O (ΔH_2_)

The overall reaction can be written as:

H_2_ +Fe^2+^ + O^2-^ +2TiO_2_ + 2H/H+/H* = 2H/H+/H* + Fe + H2O + Ti_2_O_3_

For Fe^2+^, at 1600°C

- (Fe^2+^ + O^2-^) + H_2_ = Fe + H_2_O (ΔH_r_,_Fe_ = -14.68 kj/mol Fe)

For TiO_2_ at 1600°C

- 2TiO_2_ + H_2_ = Ti_2_O_3_ + H_2_O (ΔH_r_,_Ti2O3_ = -37.04 kj/mol Ti_2_O_3_)

Therefore, the energy required to produce 442 kg of Fe (7914.76 mol) of Fe:

7914.76 mol Fe * (-14.68 kj/mol Fe) = **-116.188 MJ**

Energy required to produce 191 kg of Ti_2_O_3_ (1328.84 mol) of Ti_2_O_3_

1328.84 mol Ti_2_O_3_ * (-37.04 kj/mol Ti_2_O_3)_ = -**49.22 MJ**

The means that the total energy consumed for chemical reactions (Er):

= (-116.188 MJ) +( -49.22 MJ) **=-165.4 MJ**

which can be translated into **-45.9 kWh.**

**Energy losses**

Assuming the same reactor configuration is employed for the reduction process, the heat losses can be taken as equivalent to those measured during carbothermic operation, amounting to **1334 kWh per ton of Titania-rich compound.** These losses primarily arise from air cooling of the hearth, water cooling of the roof and walls, and the power consumption of auxiliary equipment. ^[2]^

Total energy

The total energy required is calculated as the sum of the energy required for heating and melting (E_m_), the energy required for the reactions (E_r_) and the energy losses (E_loss_).

E_T_= E_m_+ E_r_+ E_loss_

E_T_ = 889.4 kWh+(-45.9 kWh) + 1334 kWh

**E_T_ =2177.5 kWh**

We find that the energy required to produce one ton of Titania rich compound via hydrogen plasma smelting reduction (HPSR) is **2177.5 kWh**, compared with **2668 kWh** for the conventional carbothermic route, with the latter representing only a current lower bound value since some of the associated follow-up strategies to deal downstream with CO_2_ are in part also very energy intense (e.g. catalytic conversion into fuels etc.). With these bounds the energy advantage of our process corresponds to an **18.4% reduction in energy demand**. Beyond the direct energy savings, HPSR yields a refined grade of metallic iron that can be directly used in steelmaking, thereby eliminating the need for energy-intense post-processing. By contrast, carbon-based reduction routes introduce impurities such as S, P, and Si via partitioning from the fossil feedstock, which necessitates additional refining steps that are both energy, and resource-intensive. While the precise energy savings associated with bypassing conventional refining are difficult to quantify and are therefore not reported here, the avoidance of these processes represents a clear additional reduction in energy consumption. Further energy costs that might have to be added on the carbothermic side are CO_2_ capture, valorisation and/or storage, all aspects emerging today in current legislative measures as additional constraints.

**Supplementary Note 3.** **CO_2_ emissions**

The CO_2_ emissions associated with the carbothermic reduction of ilmenite for the production of Titania-rich compounds stem predominantly from two sources: approximately 82% from electricity consumption and 18% from carbon-based reduction reactions. ^[5]^ Implementation of the hydrogen plasma smelting reduction (HPSR) process enables an 18.4% reduction in energy demand, corresponding to a 15% decrease in CO_2_ emissions attributable to electricity use. In parallel, replacing carbon with green hydrogen as the reductant fully abates the 18% of emissions previously associated with the chemical reduction step. Together, these modifications result in a 34% net reduction in total CO_2_ emissions, comprising a 15% contribution from improved energy efficiency and a 19% contribution from the complete decarbonization of the reductant.

However, the proposed pathway for production of Titania-rich compound and high purity iron assumes complete reliance on renewable electricity, with carbon reductants replaced entirely by green hydrogen. By using this approach, we are able to quantify the CO_2_ emissions of our process as zero.

References

[1] P. P. Chris, Scandinavian Journal of Metallurgy **2002**, *31*, 120.

[2] F. X. Han, T. Lei, L. Zhou, Adv Mat Res **2012**, *512*, 2343.

[3] P.C. Pistorius, in the 6th International Heavy Minerals Conference ‘Back to Basics’, **2007**.

[4] S. D. R, P. H, JANAF Thermochemical Tables, 2nd ed, Washington (DC) **1971**.

[5] Sovereign Metals Limited, Life cycle assessment with overall global warming potential of upgrading ilmenite to Titania slag and synthetic rutile, 2021.
